# Acidic melanoma microenvironment selects for a senescence-like but also migratory-active subpopulation driving metastatic disease

**DOI:** 10.1038/s41420-025-02806-0

**Published:** 2025-10-20

**Authors:** Chafia Chiheb, Stefan Fischer, Zubeir El Ahmad, Ingmar Henz, Ines Böhme-Schäfer, Melanie Kappelmann-Fenzl, Anja Katrin Bosserhoff

**Affiliations:** 1 Institute of Biochemistry, Friedrich-Alexander-Universität Erlangen-Nürnberg (FAU), Erlangen, Germany; 2 Faculty of Computer Science, Deggendorf Institute of Technology, Deggendorf, Germany; 3 Department Hamm 2, Hochschule Hamm-Lippstadt, Hamm, Germany; 4 CCC WERA: Comprehensive Cancer Center Alliance WERA (CCC WERA), Erlangen, Germany; 5 BZKF: Bavarian Cancer Research Center (BZKF), Erlangen, Germany

**Keywords:** Melanoma, Senescence, Cancer microenvironment, Metastasis

## Abstract

One of the main characteristics of solid tumors, such as melanoma, is an acidic tumor microenvironment. Due to dysregulation of the cancer cell metabolism and an increased production of acidic metabolites, the tumor acidifies its microenvironment. We hypothesize that this has a strong impact on tumor heterogeneity and the formation of phenotypic subpopulations. Cell culture experiments are usually carried out at a physiological pH of 7.4. Here, we show that long-time acidosis results in the formation of a senescent subpopulation in melanoma cells. Interestingly, after reintroduction to physiological pH, these cells lose their senescence-associated attributes. We isolated this subpopulation using β-galactosidase-dependent C_12_FDG staining and FACS. Live cell imaging, microscopy, and RNA sequencing analysis revealed that these apparent senescent cells show an increased migratory activity. With this study, we demonstrate that the acidic tumor microenvironment drives tumor plasticity in melanoma and results in the formation of a small subpopulation, which promotes metastasis.

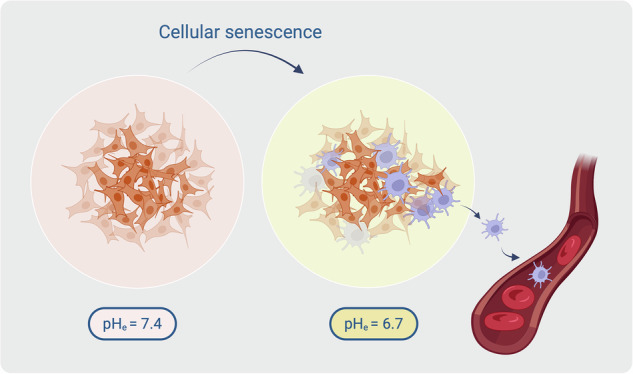

## Introduction

The incidence of malignant melanoma has been rising for years [1]. Melanoma cells exhibit significant phenotypic plasticity, complicating the development of adequate therapeutic strategies and further promoting therapy resistance. Therefore, melanoma is a crucial focus of ongoing research [2].

The deregulation of the cancer cell metabolism through upregulation of glycolysis and the increased production of acidic metabolites, such as lactate, are the reasons for the acidification of the tumor microenvironment [3]. Generally, the extracellular pH (pH_e_) of the tumor ranges from 5.8 to 7.2 [4, 5]. This is different from the physiological pH of 7.4, which is also used in in vitro cultivation of cells [6]. To closely mimic the real tumor conditions, it is essential to adjust the pH of the media to an acidic range. The effects of an acidic tumor microenvironment on human melanoma have already been the subject of investigation by our group, and we demonstrated the formation of a senescence-like phenotype as a consequence of an acidic extracellular pH [7].

Cellular senescence was first described by Hayflick and Moorhead in 1961 and is considered one of the hallmarks of ageing. They demonstrated that normal human diploid fibroblasts stop to proliferate after a certain amount of cell passages, called the Hayflick limit [8, 9]. Senescence is described to be an irreversible state with cells staying in cell cycle arrest [8, 10]. Senescent cells can only be determined by a combination of molecular markers. It was shown that one marker alone is not sufficient to reliably identify a senescent state, potentially as there are other related cellular phenotypes with corresponding molecular effects and shared markers [11]. Senescent and quiescent cancer cells both share the characteristic G_0_/G_1_-phase cell cycle arrest, in which cancer cells stop dividing. Dormant cancer cells also share this attribute, but are capable of re-entering the cell cycle if certain environmental conditions change, consequently favoring tumor relapse and metastatic disease [12, 13]. Despite the association with a permanent cell-cycle arrest, speculations of a possible reversibility of senescence have been arising for some time [12]. This would explain the ongoing overlap between definitions and markers of the described related phenotypes.

The influence of the tumor microenvironment on melanoma plasticity has not been studied, and it is open whether acidosis is a major contributor to the formation of cell subpopulations within melanoma. In this study, we give a detailed insight into the acidosis-induced senescent phenotype and compare it to non-acidosis-treated aging-related senescent cells by comprehensive molecular analysis. We present a reliable method to identify and isolate this specific cell subpopulation for a targeted investigation. By reintroducing (RE) former acidosis-treated cells back to physiological pH, we can show that tumor acidosis plays an important part in tumor cell plasticity since the observed senescence phenotype can be reversed by that. In summary, our results display how microenvironmental acidosis acts as a driver for tumor progression and plasticity as it favors the formation of a small, aggressive subpopulation, which can further drive metastatic disease.

## Results

### Physiological acidosis system triggers senescence

**Fig. 1:**
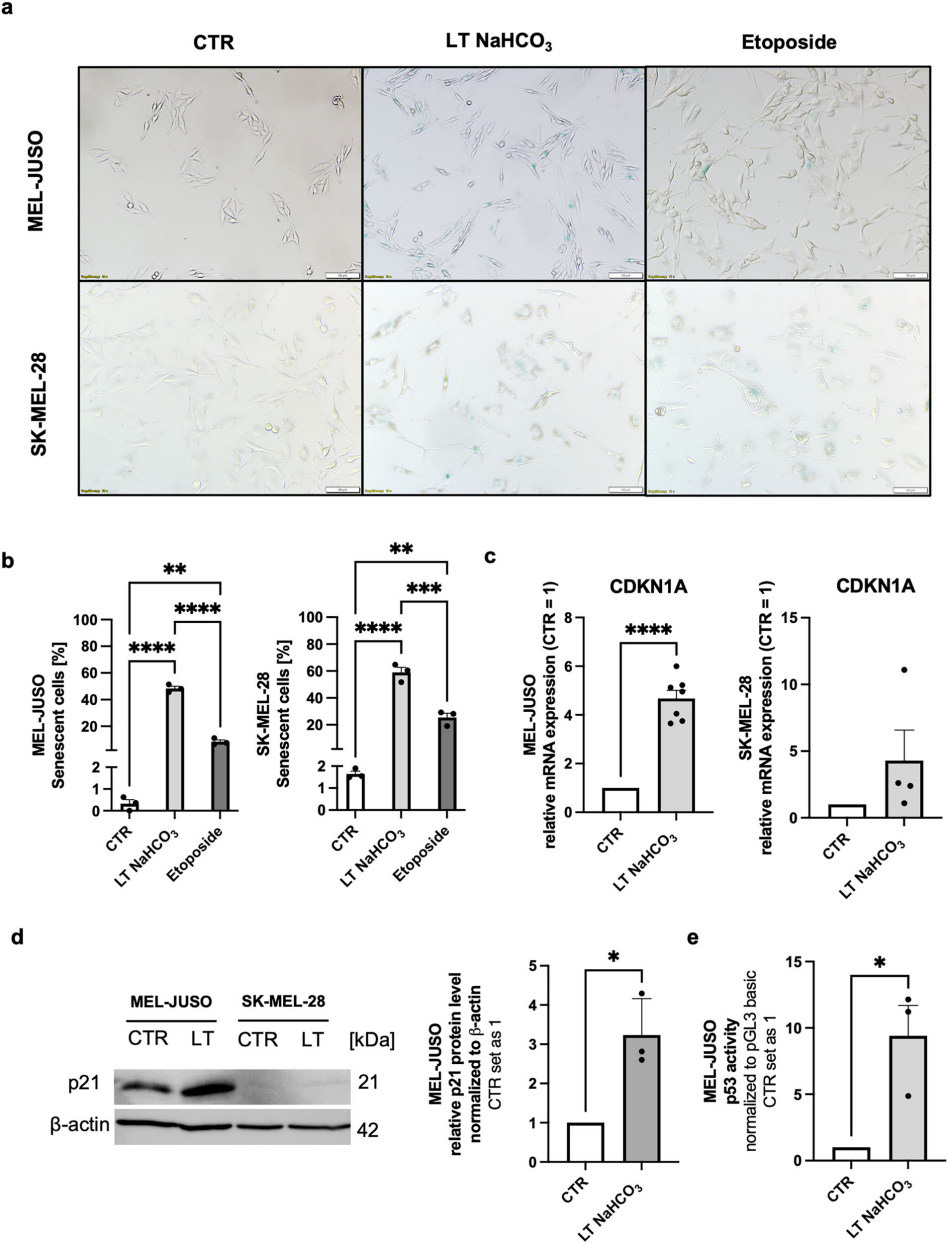
Extracellular acidosis using NaHCO_3_ buffer induces a senescence-like phenotype in melanoma cells. **a** SA-β-Gal staining of MEL-JUSO and SK-MEL-28 cell lines after LT acidosis treatment at pH 6.7 (LT NaHCO_3_) compared to cells cultivated at pH 7.4 (CTR). Etoposide-treated cells were used as a positive control. Scale bars equal 100 µm. **b** Quantification of SA-β-Gal staining (*n* = 3). Percentage of SA-β-Gal positive cells (blue) were counted in comparison to the total cell number. Results are shown as mean ± SEM (range). Statistical analysis was made using one-way ANOVA followed by Tukey’s HSD post hoc test. **c** Relative CDKN1A mRNA expression of MEL-JUSO (*n* = 7) and SK-MEL-28 (*n* = 4) cell lines after LT acidosis treatment at pH 6.7 (LT NaHCO_3_) compared to cells cultivated at pH 7.4 (CTR). Relative mRNA expression levels were normalized to β-actin expression levels. Results are shown as mean ± SEM (range). Statistical analysis was made using Student’s unpaired *t*-test. **d** Western blot of p21 protein expression (*n* = 3) after LT acidosis treatment at pH 6.7 (LT NaHCO_3_) compared to cells cultivated at pH 7.4 (CTR). Relative protein level was normalized to β-actin. Results are shown as mean ± SEM (range). Statistical analysis was made using Student’s unpaired t-test. Uncropped version in Supplement Fig. 2. **e** Luciferase-assay depicting enhanced p53 activity of MEL-JUSO (*n* = 3) cell line after LT acidosis treatment at pH 6.7 (LT NaHCO_3_) compared to cells cultivated at pH 7.4 (CTR). Results are shown as mean ± SEM (range). Statistical analysis was made using Student’s unpaired *t*-test. (*: *p* < .05, **: *p* < .01, ***: *p* < .001, ****: *p* < .0001).

The formation of a senescence-like cellular phenotype under the influence of an acidic microenvironment has already been shown by our group using a 2-(N-morpholino) ethanesulfonic acid (MES)-based buffer system to stabilize the media conditions at an extracellular pH of 6.7 [7]. Since this was an artificial system, we aimed at capturing the real tumor conditions as close as possible and decided to establish a system using the physiological buffer sodium bicarbonate for long-term treatment (LT NaHCO_3_). Long-time acidosis treatment of the cell lines MEL-JUSO, originating from primary melanoma, and SK-MEL-28, from melanoma metastasis, resulted in morphological changes compared to the respective control cells cultivated at pH_e_ = 7.4 (Fig. 1a). The cells developed larger and more granular cell bodies using the NaHCO_3_ buffer system, resembling the cell morphology, which was also described by using the MES buffer [7]. Analysis of senescence-associated β-galactosidase (SA-β-Gal) activity indicated the induction of a senescent subpopulation of about 50–60% in these acidosis-treated cells (Fig. 1a, b). Further analysis of gene expression using quantitative real-time PCR (qRT-PCR) demonstrated an increased expression of the cell cycle inhibitor p21^CIP1/WAF1^ (CDKN1A) for both cell lines (Fig. 1c). Protein expression was examined using western blotting and confirmed the data (Fig. 1d). Weaker expression in the cell line SK-MEL-28 is due to a p53 mutation [14]. In addition, increased activity of p53 as an inductor of p21 after LT acidosis treatment was confirmed by using a dual-luciferase reporter (DLR™) assay system in the cell line MEL-JUSO (Fig. 1e).

### Reversibility of the senescent phenotype depends on pH_e_

**Fig. 2:**
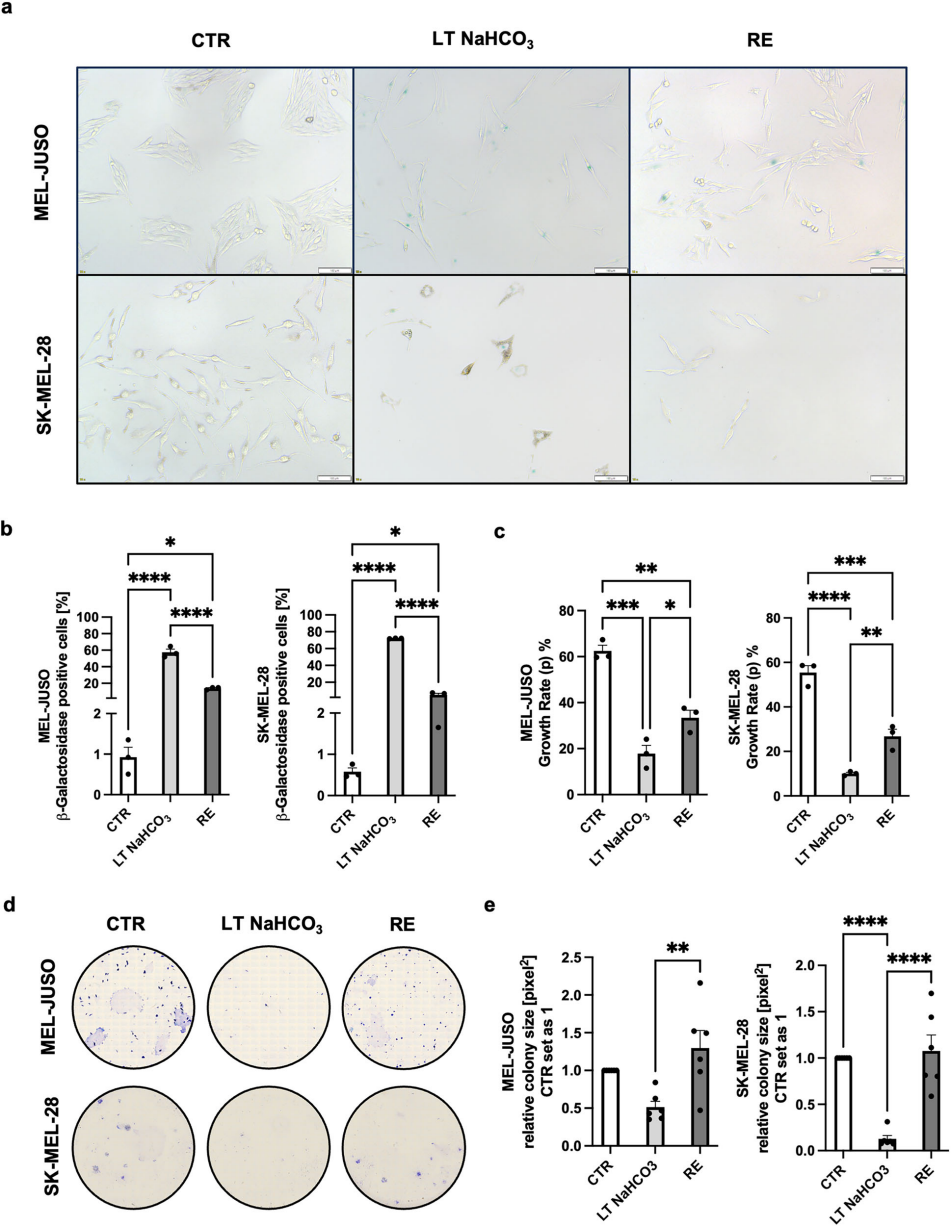
Reintroduction of long-time acidosis treated cells to physiological pH results in loss of senescent phenotype and resumption of proliferation. **a** SA-β-Gal staining of MEL-JUSO and SK-MEL-28 cell lines after reintroduction to physiological pH 7.4 after LT acidosis treatment at pH 6.7 (RE) compared to LT acidosis-treated cells (LT NaHCO_3_) and cells cultivated at pH 7.4 (CTR). Scale bars equal 100 µm. **b** Quantification of SA-β-Gal staining (*n* = 3). Percentage of SA-β-Gal positive cells (blue) were counted in comparison to the total cell number. Results are shown as mean ± SEM (range). Statistical analysis was made using one-way ANOVA followed by Tukey’s HSD post hoc test. **c** Percentage growth rate (p) of MEL-JUSO (*n* = 3) and SK-MEL-28 (*n* = 3) cell lines after reintroduction to physiological pH 7.4 after LT acidosis treatment at pH 6.7 (RE) compared to LT acidosis-treated cells (LT NaHCO_3_) and cells cultivated at pH 7.4 (CTR). Results are shown as mean ± SEM (range). Statistical analysis was made using one-way ANOVA followed by Tukey’s HSD post hoc test. **d** Pictures after colony staining of MEL-JUSO and SK-MEL-28 cell lines after reintroduction to physiological pH 7.4 after LT acidosis treatment at pH 6.7 (RE) compared to LT acidosis-treated cells (LT NaHCO_3_) and cells cultivated at pH 7.4 (CTR). In this case, reintroduction to physiological pH was carried out for 14 d prior to fixation and staining of colonies. Results are shown as mean ± SEM (range). Statistical analysis was made using one-way ANOVA followed by Tukey’s HSD post hoc test. **e** Relative colony size [pixel^2^] of MEL-JUSO (*n* = 6) and SK-MEL-28 (*n* = 6) cell lines after reintroduction to physiological pH 7.4 after LT acidosis treatment at pH 6.7 (RE) compared to LT acidosis-treated cells (LT NaHCO_3_) and cells cultivated at pH 7.4 (CTR). CTR set as 1. Results are shown as mean ± SEM (range). Statistical analysis was made using one-way ANOVA followed by Tukey’s HSD post hoc test. (*: *p* < .05, **: *p* < .01, ***: *p* < .001, ****: *p* < .0001).

Cellular senescence is described as a state of irreversible cell cycle arrest due to, e.g., impaired DNA damage response, loss of telomeres, etc. [8, 10]. Contrary to this statement, here, reintroduction (RE) of the LT NaHCO_3_-treated cells (pH_e_ = 6.7) into medium with physiological pH (pH_e_ = 7.4) for 96 h resulted in a reversal of the senescent phenotype. While morphological changes like the enlarged cell bodies were retained, SA-β-Gal activity was significantly reduced (Fig. 2a, b). Proliferation was reinstated, confirmed by measuring the growth rate by live cell imaging using 2D time-lapse inverted microscopy (Fig. 2c). Growth rates (p) of CTR cells were only partially reached in the RE cells, however, proliferation was only determined as early as 96 h after reintroduction to pH_e_ = 7.4 and might need longer observation. Cell growth was additionally assessed by clonogenic assay after reintroducing acidic-treated cells to the physiological pH of 7.4 for 14 days, and even resulted in a slightly higher proliferative activity than in CTR cells (Fig. 2d, e). The increased proliferative potential after the long-term observation of 14 days, which strongly exceeded the reestablished proliferation after 96 h observed before, indicated a time dependency of the cells to adjust to a new microenvironment and resume to proliferate. In conclusion, these data imply that LT NaHCO_3_ treatment results in a senescence-like phenotype in human melanoma cells, which differs from the classic definition of cellular senescence and therefore requests further investigation.

### Investigation of a senescence-like melanoma subpopulation

**Fig. 3:**
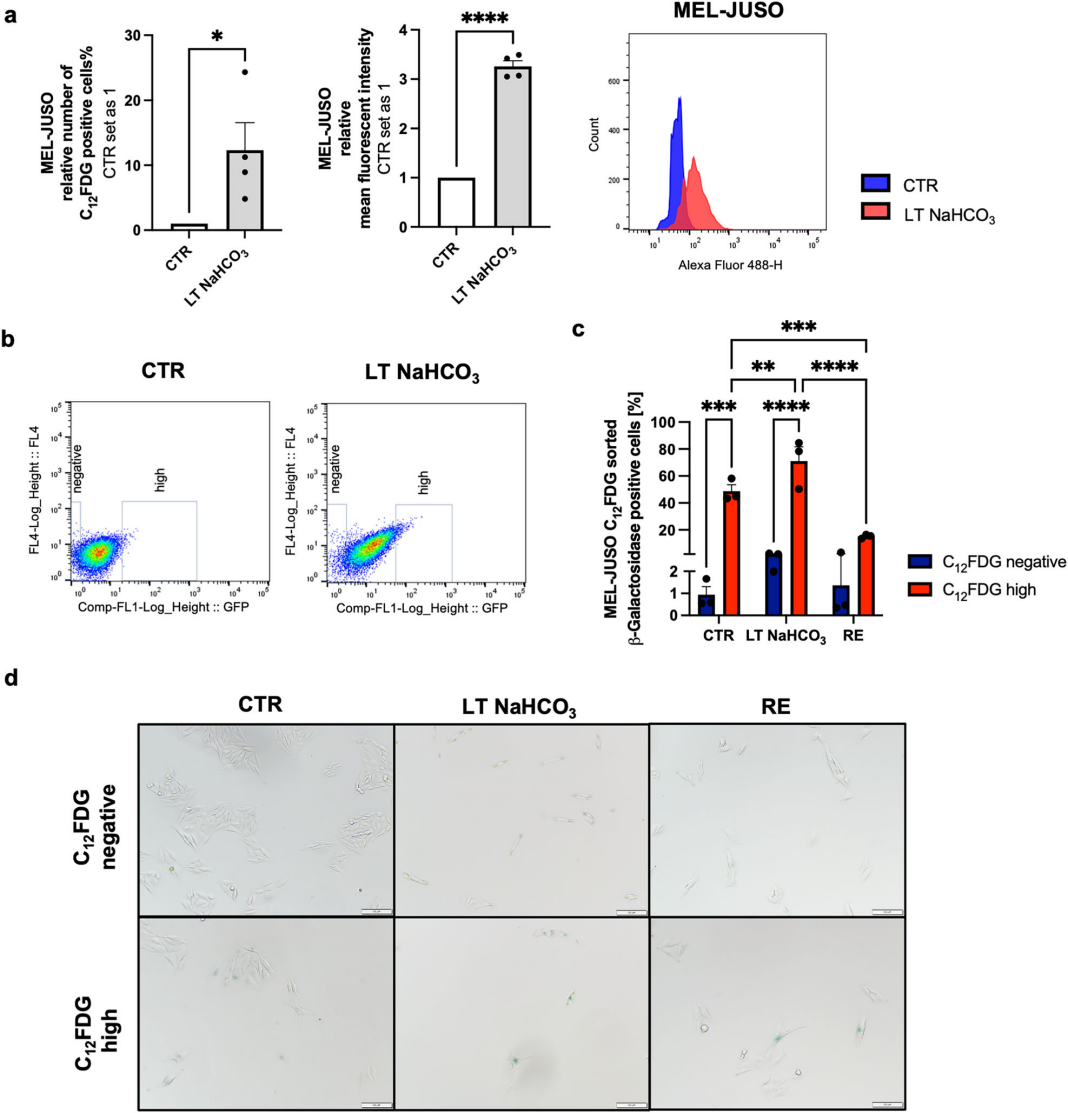
Flow cytometric analysis and isolation of senescent subpopulations with C_12_FDG staining. **a** Relative number of C_12_FDG positive MEL-JUSO cells (*n* = 4) with relative mean fluorescent intensity (middle panel) and plot (right panel) after LT acidosis treatment at pH 6.7 (LT NaHCO_3_) compared to cells cultivated at pH 7.4 (CTR). Results are shown as mean ± SEM (range). Statistical analysis was made using Student’s unpaired *t*-test. **b** Dot plots depicting the gating strategy of C_12_FDG stained MEL-JUSO CTR and LT NaHCO_3_ cells for fluorescence-activated cell sorting (FACS). **c** Quantification of SA-β-Gal staining of MEL-JUSO subpopulations after FACS (*n* = 3). Percentage of SA-β-Gal positive cells (blue) were counted in comparison to the total cell number. Results are shown as mean ± SEM (range). Statistical analysis was made using two-way ANOVA. **d** SA-β-Gal staining of MEL-JUSO subpopulations after C_12_FDG FACS. Scale bars equal 100 µm. (*: *p* < .05, **: *p* < .01, ***: *p* < .001, ****: *p* < .0001).

For an in-depth analysis of the LT NaHCO_3_-induced senescent phenotype, we used β-galactosidase-dependent 5-dodecanoylaminofluorescein di-β-D-galactopyranoside (C_12_FDG) staining to isolate the senescent subpopulation by fluorescent-activated cell sorting (FACS) [15]. C_12_FDG staining of the cell line MEL-JUSO and analysis by flow cytometry confirmed prior results of increased SA-β-galactosidase activity in LT NaHCO_3_ acidosis-treated whole cell population (Fig. 3a). Subsequently, the highest (C_12_FDG^high^) and lowest (C_12_FDG^negative^) two percent of C_12_FDG-stained CTR and LT NaHCO_3_-treated cells were isolated by FACS (Fig. 3b). Conventional SA-β-Gal staining using X-Gal was used to determine the sorting efficiency and showed an enrichment of >50% β-Gal positive cells in the CTR C_12_FDG^high^ population, which normally only makes up about two percent of the whole cell population (Fig. 3c, d). After sorting, LT NaHCO_3_ subpopulations were reintroduced to pH_e_ = 7.4 (RE) for 96 h. This resulted in a strong decrease of senescent cells in the C_12_FDG^high^ population from >70% to only 15% SA-β-Gal positive cells and confirmed the results obtained from the whole cell populations, supporting a reversible pH-dependent senescent phenotype in acidosis-treated melanoma cells.

### Transcriptome-based analysis of senescent phenotype

To further characterize the discovered senescent phenotype, we conducted RNA-sequencing (RNA-seq) of the C_12_FDG^negative^ and C_12_FDG^high^ (CTR and LT NaHCO_3_, respectively) subpopulations and performed differential gene expression analysis. First, an enrichment analysis via the *gene set enrichment analysis* (GSEA) tool based on the RNA-seq read counts normalized to library size was carried out in 19 gene sets containing the term “senescence” in the gene set name from the Molecular Signature Database (MSigDB, Version 2023) to confirm the senescent phenotype. Hence, comparing CTR C_12_FDG^high^ cells with CTR C_12_FDG^negative^ cells, the analysis clearly revealed a senescence signature of the CTR C_12_FDG^high^ cells (Table 1). This subpopulation showed an enrichment of classic senescence gene sets, such as Saul_Sen_Mayo [16] and Fridman_Senescence_up, in which 121 of 125 and 74 of 77 genes, respectively, of the corresponding gene set are induced. Additionally, the CTR C_12_FDG^high^ cells revealed an enrichment of two gene sets from WikiPathway (WP), namely Sphingolipid_Metabolism_in_Senescence and Prostaglandin_and_Leukotriene_Metabolism_in_Senescence, in which 27 of 28 and 28 of 31 genes are expressed, respectively. The corresponding senescence signature could also be confirmed in the LT NaHCO_3_ C_12_FDG^high^

**Table 1:** GSEA: Significant enriched gene sets from GSEA of CTR C12FDG^high^ vs CTR C12FDG^negative^.

CTR C12FDG^high^ vs CTR C12FDG^negative^	enriched in CTR C_12_FDG^high^		
Gene set	Size	NES	FDR
SAUL_SEN_MAYO	121	2,04	0,005
FRIDMAN_SENESCENCE_UP	74	1,98	0,002
WP_SPHINGOLIPID_METABOLISM_IN_SENESCENCE	27	1,33	0,193
WP_PROSTAGLANDIN_AND_LEUKOTRIENE_METABOLISM_IN_SENESCENCE	28	1,32	0,158

	enriched in CTR C_12_FDG^negative^		
Gene set	Size	NES	FDR
N/A			

cells compared to LT NaHCO_3_ C_12_FDG^neg^, in which GSEA revealed an enrichment of the same senescence gene sets as in the CTR C_12_FDG^high^ cells except of Sphingolipid_Metabolism_in_Senescence (Table 2). However, we surprisingly observed an enrichment of other senescence gene sets in the LT NaHCO_3_ C_12_FDG^negative^ cell population compared to the LT NaHCO_3_ C_12_FDG^high^ population (Table 2), namely Oncogene_Induced_Senescence (33 of 37 genes expressed), Cellular_senescence (128 of 213 genes expressed), and Oxidative_Stress_Induced_Senescence (64 of 133 genes expressed). These gene sets have only a very small overlap with the gene sets that are enriched in both LT and CTR cultivated C_12_FDG^high^ cells (Fig. 4a).

**Fig. 4:**
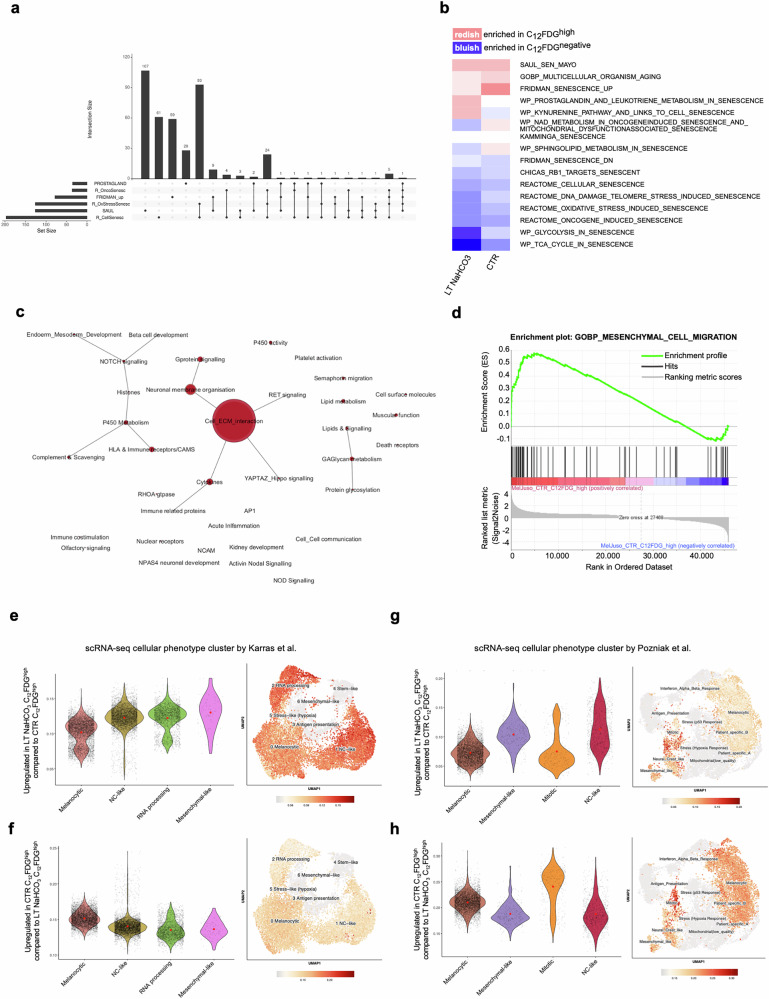
RNA-sequencing analysis of sorted subpopulations. **a** Intersection of enriched senescence gene sets from MSigDB. An UpSet plot shows a very high intersection of the senescence gene sets from the Reactome database, whereas the other gene sets, which are enriched in C_12_FDG^high^ cells, are mainly composed of unique genes. Each line represents a gene set. The bars above indicate the size of each set or intersection. Intersections are shown through connecting lines. **b** mitch-analysis after RNA-Seq of MEL-JUSO subpopulations. Gene set enrichment by mitch using senescence-related gene sets from the “Molecular Signatures Database” (MSigDB). Heatmap depicting gene sets enriched in C_12_FDG^negative^ (blue) and C_12_FDG^high^ cells (red) in CTR and LT NaHCO_3_ conditions (right and left column, respectively). **c** Enrichment map. GSEA analysis using canonical pathway gene sets of MSigDB (C2cp) comparing CTR C_12_FDG^high^ (pH 7.4) and LT NaHCO_3_ C_12_FDG^high^ subpopulations shows enrichment of gene sets associated to cell-matrix interaction after long-time acidosis treatment of melanoma cells. The size of the node represents the number of gene sets summarized inside. **d** Enrichment plot: GOBP_MESENCHYMAL_CELL_MIGRATION illustrating the profile of the running enrichment score (green) and position of the enriched gene set and the rank-ordered list of genes differentially expressed of GSEA analysis of MEL-JUSO C_12_FDG^high^ subpopulations. **e** Assignment of DEGs comparing C_12_FDG^high^ subpopulations in CTR and LT NaHCO_3_ with published scRNA-seq data of malignant melanoma [18]. The cell state annotated marker genes described by Karras et al. were used to match the LT NaHCO_3_ C_12_FDG^high^ and CTR C_12_FDG^high^ **(f)** specific gene signature composed of upregulated genes (LFC > 1.5) with the validated clusters in a melanoma mouse model. **g** Assignment of DEGs comparing C_12_FDG^high^ subpopulations in CTR and LT NaHCO_3_ with published scRNA-seq data of malignant melanoma [19]. The cell state annotated marker genes described by Pozniak et al. were used to match the LT NaHCO_3_ C_12_FDG^high^ and CTR C_12_FDG^high^ **(h)** specific gene signature composed of upregulated genes (LFC > 1.5) with the validated clusters in human melanoma tumor biopsy samples.

**Table 2:** GSEA: Significant enriched gene sets from GSEA of LT NaHCO_3_ C_12_FDG^high^ vs LT NaHCO_3_ C_12_FDG^negative^.

LT NaHCO_3_ C_12_FDG^high^ vs LT NaHCO_3_ C_12_FDG^negative^	enriched in LT NaHCO_3_ C_12_FDG^high^		
Gene set	Size	NES	FDR
WP_PROSTAGLANDIN_AND_LEUKOTRIENE_METABOLISM_IN_SENESCENCE	28	1,94	0,001
FRIDMAN_SENESCENCE_UP	73	1,78	0,015
SAUL_SEN_MAYO	121	1,76	0,011

	enriched in LT NaHCO_3_ C_12_FDG^negative^		
Gene set	Size	NES	FDR
REACTOME_ONCOGENE_INDUCED_SENESCENCE	33	-1,24	0,201
REACTOME_CELLULAR_SENESCENCE	128	-1,40	0,117
REACTOME_OXIDATIVE_STRESS_INDUCED_SENESCENCE	64	-1,54	0,086

As illustrated in Table 1 and Table 2, the gene sets linked to senescence enriched in CTR C_12_FDG^high^, LT NaHCO_3_ C_12_FDG^high^, and LT NaHCO_3_ C_12_FDG^negative^ cells differ. The observed enrichment of senescent gene sets in LT NaHCO_3_ C_12_FDG^negative^ cells might be based on a broader senescence-relevant signature differing from the highly defined and validated signature described by Saul et al. [16].

To further identify the effect of an acidic environment on the senescence-associated gene pattern, a direct comparison of C_12_FDG^high^ and C_12_FDG^negative^ cells cultivated under CTR or LT NaHCO_3_ conditions, respectively, was conducted by *mitch*, a multi-dimensional enrichment analysis using a rank-MANOVA statistical test [17], allowing to compare more than two sample groups at once. The analysis of C_12_FDG^high^ (Fig. 4b; colored redish) in contrast to C_12_FDG^negative^ cells (Fig. 4b; colored bluish) in LT NaHCO_3_ and CTR treated cells (Fig. 4b; left and right column) resulted in a common senescent signature in both conditions, LT NaHCO_3_ and CTR treated. The observed enrichment with regards to the Saul_Sen_Mayo gene set [16], indicates a treatment-independent basic senescent phenotype and is thus constantly enriched in C_12_FDG^high^ CTR as well as LT NaHCO_3_-treated cells. This general phenotype is accompanied by a varying degree of enrichment of the gene sets of Multicellular_Organism_Aging and Fridman_Senescence_up in the CTR and LT NaHCO_3_ C_12_FDG^high^ populations, already indicating differences between the two conditions. Moreover, in accordance with the GSEA results, the senescence gene sets from the Reactome database are enriched in C_12_FDG^negative^ cells in both conditions, which in turn suggests a reduced importance of these genes for the senescent phenotype of C_12_FDG^high^ cells. However, the enrichments comparing the C_12_FDG^negative^ CTR and LT NaHCO_3_ treated cells show a clear difference in their Enrichment-Score (s-value; illustrated as color intensity in the enrichment heatmap). Collectively, the enrichment analyses via GSEA and *mitch* indicate a similar senescence signature in either CTR or LT NaHCO_3_ C_12_FDG^high^ subpopulations based on the Saul_Sen_Mayo gene set compared to the corresponding C_12_FDG^negative^ ones. Additionally, the multidimensional analyses confirm the GSEA analysis, but newly indicate an acidosis-induced difference of the senescence phenotype of C_12_FDG^high^ and C_12_FDG^negative^ cells.

For a more detailed classification of the observed phenotype comparing both culture conditions of the C_12_FDG^high^ subpopulation, we first performed gene set enrichment analysis via GSEA using the canonical pathway gene set collection of the MSigDB (C2cp). This analysis showed a high enrichment of gene sets associated to cell-matrix interaction in the LT NaHCO_3_ C_12_FDG^high^ cells compared to CTR NaHCO_3_ C_12_FDG^high^ (Table 3, Fig. 4c).

**Table 3:** GSEA: Top 10 of all significant enriched gene sets in LT NaHCO_3_ C_12_FDG^high^ vs CTR NaHCO_3_ C_12_FDG^high^ cells using canonical pathway gene set collection of the MSigDB (C2cp).

Gene set	Size	NES	FDR
KEGG_ECM_RECEPTOR_INTERACTION	83	2,88	0
NABA_CORE_MATRISOME	263	2,86	0
PID_INTEGRIN1_PATHWAY	66	2,8	0
REACTOME_EXTRACELLULAR_MATRIX_ORGANIZATION	297	2,75	0
REACTOME_LAMININ_INTERACTIONS	30	2,71	0
REACTOME_ECM_PROTEOGLYCANS	75	2,68	0
NABA_BASEMENT_MEMBRANES	40	2,66	0
NABA_ECM_GLYCOPROTEINS	186	2,63	0
REACTOME_DEGRADATION_OF_THE_EXTRACELLULAR_MATRIX	139	2,6	0
REACTOME_COLLAGEN_FORMATION	90	2,59	0

Additionally, using the gene set collection of the gene ontology biological process (GOBP), the gene set Mesenchymal_Cell_Migration showed a significant enrichment among the 20 most significantly enriched gene sets (Fig. 4d, Supplement Table 1), surprisingly indicating an increased expression of migratory-related genes in LT NaHCO_3_ C_12_FDG^high^ cells. This hints to an impact of LT acidosis treatment on plasticity of the melanoma cells.

A study by Karras et al. provided proof of a hierarchical model in melanoma that uncouples tumor growth and metastasis by combining mouse genetics, single-cell and spatial transcriptomics, lineage tracing and quantitative modeling to grasp the magnitude of cell-state diversity in melanoma. The data show that the ability for metastasis is limited to a distinct subpopulation with a mesenchymal-like cell state. These cells were found to be in the periphery of the tumor, not contributing to tumor growth, while cells attributed to fuel tumor growth have been mainly assigned to the center of the tumor [18]. Similar subtypes were published by the same group using scRNA-seq data of human melanoma metastatic biopsies, which further demonstrates melanoma heterogeneity and plasticity [19]. Overrepresentation analyses revealed a strong association of genes upregulated in LT NaHCO_3_ C_12_FDG^high^ with mesenchymal and neural crest-like gene signatures, while upregulated genes in CTR C_12_FDG^high^ showed a significant enrichment within the melanocytic and mitotic subclusters defined by Karras et al. (Fig. 4e, f; Supplement Fig. 3a) or Pozniak et al. (Fig. 4g, h; Supplement Fig. 3b). Further, analyses of the expression status of the defined melanocytic, RNA processing or mitotic, NC-like and mesenchymal cell type cluster by Karras et al. or Pozniak et al., respectively, were performed to validate the annotation with the defined clusters (Supplement Fig. 3c, d). The majority of genes defined by Pozniak et al. annotated to the mitotic cell cluster are predominantly downregulated in LT NaHCO_3_ C_12_FDG^high^ cells and those annotated to the neural crest-like and mesenchymal cell clusters upregulated compared to the gene expression of CTR C_12_FDG^high^ cells (Supplement Fig. 3d). Further, the RNA-seq data revealed a central transcriptional regulator, mesenchymal-like marker transcription factor 4 (TCF4), to be significantly induced in LT C_12_FDG^high^ subpopulation compared to CTR C_12_FDG^high^ (log2FC 3.24, p_adj_ 1.32 × 10^−4^). This hints to the underlying molecular mechanistics of the phenotype switch after LT acidosis treatment.

### Migratory active subpopulation in human melanoma

**Fig. 5:**
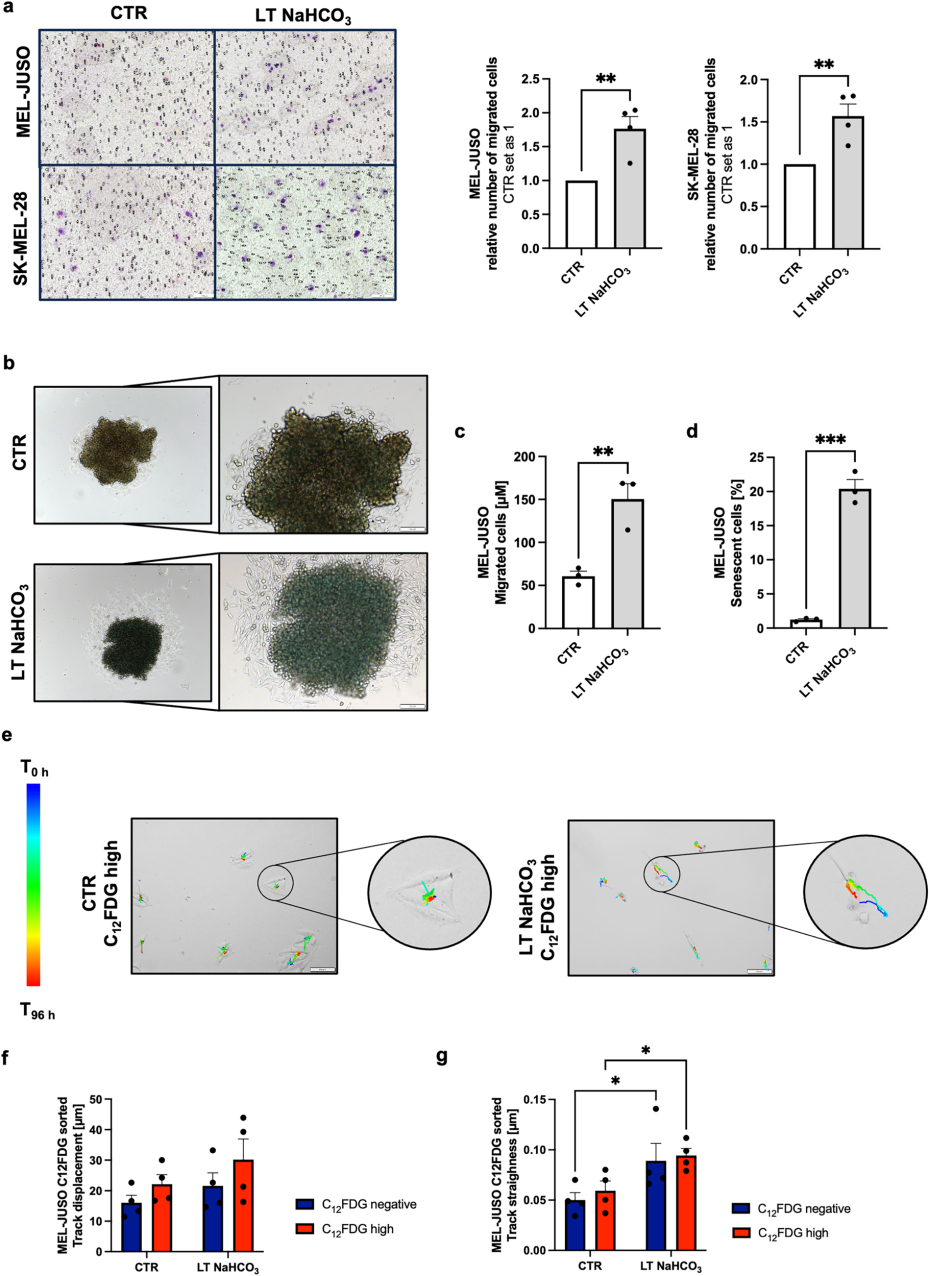
Analysis of migratory phenotype after LT acidosis treatment of melanoma cells. **a** Boyden chamber assay (*n* = 4) of MEL-JUSO and SK-MEL-28 cell lines after LT acidosis treatment at pH 6.7 (LT NaHCO_3_) compared to cells cultivated at pH 7.4 (CTR). Number of migrated cells (violet) was counted at 10x magnification and normalized to number of cells migrated in CTR cells. Results are shown as mean ± SEM (range). Statistical analysis was made using Student’s unpaired *t*-test. **b** Pictures of SA-β-Gal stained spheroids generated from MEL-JUSO cell line after LT acidosis treatment at pH 6.7 (LT NaHCO_3_) compared to spheroids from cells cultivated at pH 7.4 (CTR). Scale bars equal 100 µm. **c** Distance measurement of cells that migrated out of the spheroids in µM (*n* = 3). Results are shown as mean ± SEM (range). Statistical analysis was made using Student’s unpaired *t*-test. **d** Quantification of SA-β-Gal staining of MEL-JUSO spheroids (*n* = 3). Percentage of SA-β-Gal positive cells (blue) were counted in comparison to the total cell number that migrated out of the spheroids. Results are shown as mean ± SEM (range). Statistical analysis was made using Student’s unpaired *t*-test. **e** Live imaging analysis of MEL-JUSO subpopulations after C_12_FDG FACS. Overlay of Tracks and T_96 h_ time frame of C_12_FDG^high^ populations of MEL-JUSO CTR and MEL-JUSO LT NaHCO_3_. **f** Live imaging analysis (*n* = 4). Track displacement of MEL-JUSO subpopulations after 96 h. Results are shown as mean ± SEM (range). Statistical analysis was made using two-way ANOVA. **g** Live imaging analysis (*n* = 4). Track straightness of MEL-JUSO subpopulations after 96 h. Results are shown as mean ± SEM (range). Statistical analysis was made using two-way ANOVA. (*: *p* < .05, **: *p* < .01, ***: *p* < .001, ****: *p* < .0001).

To validate the induction of a migratory phenotype under long-time exposure to an acidic tumor microenvironment, we first performed Boyden chamber assays of MEL-JUSO and SK-MEL-28 whole cell populations and revealed an increase in direct migration in LT NaHCO_3_ treated cells compared to their CTR cells (Fig. 5a). Live cell imaging using 2D time-lapse inverted microscopy of the whole populations showed decelerated attachment capacity and therefore a longer time span for the cell spreading of acidosis-treated cells compared to CTR cells cultivated at pH_e_ 7.4 (Supplement Fig. 4a). However, tracking analysis revealed no track displacement, which specifies the distance between start point and end point and no overall track straightness, which indicates the ratio between the track length and displacement of the traveled object, in LT NaHCO_3_ and CTR whole cell populations (Supplement Fig. 4b, c). To capture the effects of microenvironmental acidosis on melanoma cells in a three-dimensional (3D) setting, we generated spheroids (Fig. 5b) from acidosis treated (LT NaHCO_3_), as well as control cells (CTR). We revealed that more cells exited the LT NaHCO_3_ spheroids than the CTR spheroids indicating a more migratory phenotype after LT acidosis treatment (Fig. 5c). Moreover, we evaluated the status of senescence by performing senescence-associated β-galactosidase staining of the spheroids. Non surprisingly the hypoxic, nutrient deprived core of the spheroids showed an obvious blue staining (β-galactosidase positive) for the spheroids from LT NaHCO_3_ and CTR cells. In addition, cells exiting and on the outer layer of the spheroids showed a significant increase in the number of blue (β-galactosidase positive) cells only for the spheroids from LT NaHCO_3_ cells (20.4%) compared to spheroids generated from control cells (1.2%) indicating for a migratory active subset of cells only after acidosis treatment (Fig. 5d). As we hypothesize that the observed effects in direct migration are due to the identified and isolated C_12_FDG^high^ subpopulation in the LT NaHCO_3_ cells and the effects are strongest in these senescence-like cells, we repeated the live cell imaging with the sorted subpopulations. Time-lapse inverted microscopy was carried out over a 96 h time period for C_12_FDG^negative^ and C_12_FDG^high^ subpopulations of CTR and LT NaHCO_3_ cells, respectively. As observed for the whole cell populations, the attachment capacity for both acidosis-treated LT NaHCO_3_ subpopulations, C_12_FDG^high^ and C_12_FDG^negative^ cells, were severely slower compared to the respective control subpopulations and by that the ability to spread in a certain time after reseeding (Supplement Fig. 4d). No difference could be observed between the C_12_FDG^high^ and C_12_FDG^negative^ subpopulations of the CTR and LT NaHCO_3_ cells, respectively, thus this effect is solely linked to the effects of the acidic microenvironment. Interestingly, tracking analysis made it possible to capture migration in C_12_FDG^high^ acidosis-treated cells (Fig. 5e), which was not detectable in the whole cell populations. Here, an increase in track displacement and track straightness of the acidosis-treated cell subpopulations was revealed (Fig. 5f, g). Hence, we were able to experimentally validate the migratory phenotype indicated by the gene set enrichment analysis of RNA-seq data of the sorted senescent subpopulations.

### Molecular reasons for migration in subpopulation

**Fig. 6:**
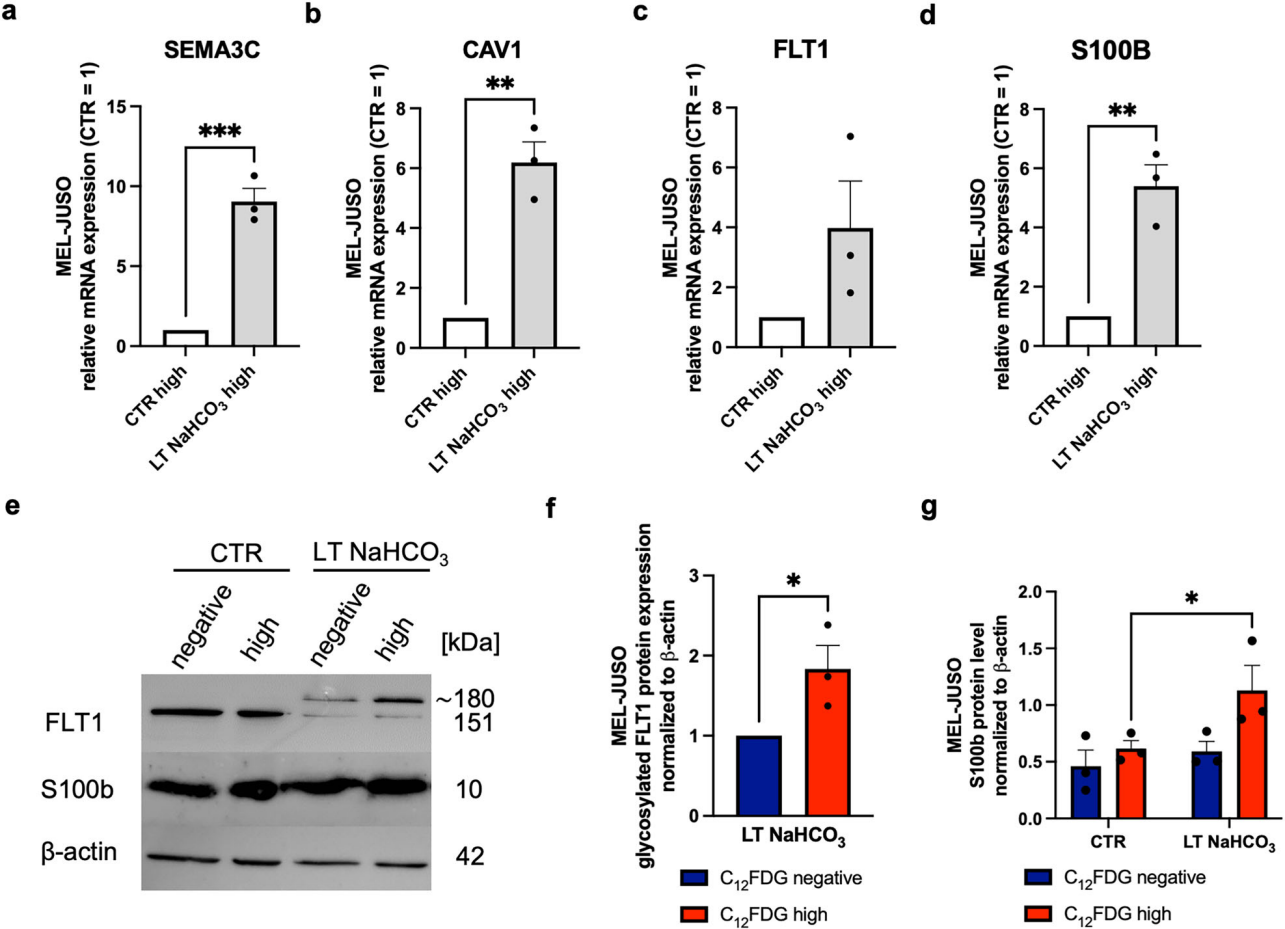
Molecular analysis of migratory phenotype in acidosis induced subpopulations of MEL-JUSO melanoma cell lines. **a** Relative SEMA3C mRNA expression (*n* = 3) of C_12_FDG^high^ populations of MEL-JUSO CTR and MEL-JUSO LT NaHCO_3_. Results are shown as mean ± SEM (range). Statistical analysis was made using Student’s unpaired *t*-test**. b** Relative CAV1 mRNA expression (*n* = 3) of C_12_FDG^high^ populations of MEL-JUSO CTR and MEL-JUSO LT NaHCO_3_. Results are shown as mean ± SEM (range). Statistical analysis was made using Student’s unpaired *t*-test**. c** Relative FLT1 mRNA expression (*n* = 3) of C_12_FDG^high^ populations of MEL-JUSO CTR and MEL-JUSO LT NaHCO_3_. Results are shown as mean ± SEM (range). Statistical analysis was made using Student’s unpaired *t*-test. **d** Relative S100B mRNA expression (*n* = 3) of C_12_FDG^high^ populations of MEL-JUSO CTR and MEL-JUSO LT NaHCO_3_. Results are shown as mean ± SEM (range). Statistical analysis was made using Student’s unpaired t-test. **e** Western blots of FLT1 and S100B protein expression of MEL-JUSO C_12_FDG sorted subpopulations after LT acidosis treatment at pH 6.7 (LT NaHCO_3_) compared to cells cultivated at pH 7.4 (CTR). Protein expression of β-actin was used as reference. Uncropped versions in Supplement Figs. 5 and 6. **f** Quantification of glycosylated FLT1 protein expression (*n* = 3) LT NaHCO_3_ MEL-JUSO C_12_FDG^high^ compared to LT NaHCO_3_ MEL-JUSO C_12_FDG^negative^ subpopulation. Relative protein level was normalized to actin. Results are shown as mean ± SEM (range). Statistical analysis was made using Student’s unpaired *t*-test. **g** Quantification of S100B protein expression (*n* = 3) of MEL-JUSO C_12_FDG sorted subpopulations after LT acidosis treatment at pH 6.7 (LT NaHCO_3_) compared to cells cultivated at pH 7.4 (CTR). Relative protein level was normalized to β-actin. Results are shown as mean ± SEM (range). Statistical analysis was made using two-way ANOVA. (*: *p* < .05, **: *p* < .01, ***: *p* < .001, ****: *p* < .0001).

To illustrate and understand the migratory aspects under LT acidosis treatment on a molecular level, we aimed at discovering the genes involved and differentially regulated compared to CTR pH 7.4 treated cells. GSEA analysis of RNA-sequencing data of the C_12_FDG^high^ populations of CTR and LT NaHCO_3_ sorted population was performed as described. We chose 16 gene sets from the GSEA results (Supplement Table 2) indicating a connection to migration and then focused on the genes illustrating significant gene expression differences (≥5-fold) and narrowed down our candidates with an extensive literature search. Finally, we performed qRT-PCR analysis of the selected genes in the LT NaHCO_3_ C_12_FDG^high^ compared to the CTR C_12_FDG^high^ population. Membrane proteins like Semaphorine 3C (SEMA3C, Fig. 6a) and other semaphorines, Caveolin-1 (CAV1, Fig. 6b) and Vascular Endothelial Growth Factor Receptor 1 (VEGFR-1/FLT1, Fig. 6c) showed an increased expression of mRNA levels in the acidosis-treated C_12_FDG^high^ cell subpopulation. These results confirmed the bioinformatic gene set enrichment analysis of the respective RNA-seq data. FLT1 protein is a 151 kDa transmembrane receptor. Post-translational modification is necessary to produce a ∼180 kDa matured glycoprotein [20, 21]. Western blot analysis of FLT1 (Fig. 6e, f) revealed the protein expression of mature FLT1 (180 kDa) after LT acidosis treatment, which is significantly upregulated in the LT NaHCO_3_ C_12_FDG^high^ subpopulation compared to their respective C_12_FDG^negative^ subpopulation. Since the expression of CAV1 is not only linked to migration but also a marker of poor prognosis [22], we wanted to additionally assess disease prognosis with analyzing expression levels of S100B gene encoding for calcium binding protein B, a prognostic marker in melanoma [23]. The mRNA expression (Fig. 6d) as well as the S100B protein level was significantly increased in acidosis-treated C_12_FDG^high^ population (Fig. 6e, g), which demonstrates the importance of the characterized subpopulation regarding therapeutic strategies.

## Discussion

Malignant melanoma is one of the most heterogenous tumor entities, being a huge burden for developing effective treatment strategies. Its high metastatic potential at early tumor stages further hampers with the clinical success of available treatment options [24–26]. The intratumoral heterogeneity has been described as a resistance mechanism of melanoma cells to treatment. The ability of melanoma cells to adapt to environmental changes and switch phenotypes adds another cause to genetic alterations being responsible for acquiring resistances against immunotherapy and targeted therapy agents, such as Vemurafenib, and therefore enables disease progression [24, 27, 28]. Many different subpopulations have been described in the last couple of years with the tumor microenvironment being an important driver for their formation [24]. In a previous study, we could show that an acidic tumor microenvironment results in the formation of senescent subpopulation with an ATF4-mediated MITF^low^/AXL^high^ phenotype using 2-(N-morpholino) ethanesulfonic acid (MES) as a buffer system [7]. To simulate real tumor conditions as closely as possible, we chose to use the physiological buffer system sodium bicarbonate (NaHCO_3_) to regulate the pH of the cell culture media. Our findings revealed that prolonged acidosis treatment of melanoma cells induces a senescence-associated phenotype. This is evidenced by the p21^CIP1/WAF1^ (CDKN1A)-mediated cell cycle arrest and the increased activity of SA-β-galactosidase.

In the classical concept, senescence is described as an irreversible cellular state [8, 10]. Interestingly, reintroducing acidosis-induced senescent cells into media with physiological pH showed that the observed effects were reversible within 4 days. During this period, SA-β-galactosidase activity significantly decreased, and calculations of the growth rate indicated a regain of proliferation. Clonogenic assays revealed a complete recovery of the proliferative capacity after 14 days of reintroduction to the physiological pH of 7.4. Similar findings of reversible senescence have been observed in melanoma in other conditions before. Sun et al. revealed a connection between the activation of TGF-β signaling and epidermal growth factor (EGFR) expression through acquired resistance to BRAF^V600E^ inhibitors. High levels of EGFR during drug treatment result in typical features of senescence, such as increased SA-β-Gal activity and p21 expression. However, when treatment was discontinued, the expression levels were reversed, and cells regained their proliferative abilities. This provides a possible explanation for why some patients regain sensitivity to drugs after treatment pauses [29]. Furthermore, Webster et al. show that WNT5A expression induces a senescence-like phenotype in melanoma but also drives invasion. Conversely, knocking down WNT5A expression reverses the observed senescence-associated effects. Since eliminating all tumor cells in vivo during therapeutic settings is not always feasible, alternative therapies aim to stop proliferation and, consequently, strive for therapy-induced senescence. However, they concluded that this reversible senescence-like phenotype, which they termed “pseudosenescence”, can develop in response to tumor treatment. Cells adapt these senescence features but remain aggressive and invasive. This finding is crucial when evaluating treatment efficiency based on well-established markers, such as p21 expression [30]. Our study confirms the existence of pseudosenescence in tumor cells. Given the controversy surrounding this topic and the lack of a unique marker to accurately define the senescence phenotype, we focused our study on isolating the acidosis-induced senescent-like subpopulation and subjecting it to intense transcriptome analysis. As we could already show, the senescence-associated phenotype was best described using SA-β-galactosidase staining [11]. Thus, we used C_12_FDG, a β-galactosidase substrate which upon cleavage results in a fluorescent product and is detectable by flow cytometry [15]. Subsequently, RNA sequencing and enrichment analysis by GSEA and *mitch* was performed resulting in a clear senescent phenotype in both, acidosis-treated (LT NaHCO_3)_, and non-acidosis-treated (CTR) C_12_FDG^high^ sorted cell subpopulations with a similar pattern of senescence markers for Saul_Sen_Mayo [16]. However, the acidosis-treated C_12_FDG^negative^ population also showed an enrichment of senescent gene sets, which concludes a general effect of acidosis on the development of a senescent phenotype, which can differ in the degree of gene expression. This is especially apparent in the degree of enriched senescent gene sets by the *mitch* analysis of CTR C_12_FDG^negative^ and LT NaHCO_3_ C_12_FDG^negative^ cells. There is only a minor overlap in the gene sets enriched in LT NaHCO_3_ C_12_FDG^high^ and LT NaHCO_3_ C_12_FDG^negative^. Therefore, it could also be assumed that both CTR C_12_FDG^high^ and LT NaHCO_3_ C_12_FDG^high^ cells, which are showing the highest β-galactosidase activity, have a robust and tissue-independent core senescence phenotype as described by Saul et al. [16]. LT NaHCO_3_ C_12_FDG^negative^ cells might only show distinct cellular characteristics, which are frequently found in senescent cells, without being a necessity for senescence. It might even be that a cell with the senescence phenotype described by Saul et al. [16], without having all or some of the other described features of senescence, can exit the senescent phenotype, contrary to a real senescent cell and only in the context of malignancy.

Further analysis between only the two senescent C_12_FDG^high^ populations showed a high enrichment of gene sets associated to cell-matrix interaction and Mesenchymal_Cell_Migration as one of the most enriched gene sets only in the cells under acidic microenvironmental conditions. This pointed to an acidosis-specific difference between the senescent subpopulations. Interestingly, the extra- and intracellular pH level has been under investigation as a main regulator for cell migration and could be shown to influence migration in many different ways [31]. Extracellular acidosis, for example, has been demonstrated to regulate epithelial-to-mesenchymal transition (EMT) [32]. Further, Estrella et al. revealed that extracellular acidosis promotes local tumor invasion, as their study confirmed a strong correlation between tumor invasiveness and the peritumoral pH value [33]. This was supported by Karras et al. by combining mouse genetics, single-cell and spatial transcriptomics, lineage tracing and quantitative modeling to provide proof of a hierarchical model in melanoma that resulted in a spatially and temporally resolved map of the diversity and trajectories of melanoma cell states. Cells in the outer periphery of the tumor exhibited a mesenchymal-like cell state that promotes migration, whereas cells that have been found spatially and functionally distant in the center of the tumor were assigned responsibility for tumor growth [18]. We mapped the significantly upregulated genes in the LT NaHCO_3_C_12_FDG^high^ subpopulation to these clusters and, additionally, to patient data-derived melanoma cell type clusters defined by Pozniak et al. [19] and could confirm the mesenchymal-like cell state in the acidosis-induced highly senescent subpopulation. This further proves the importance of tumor acidosis in driving melanoma metastasis. Several studies have already shown the link between tumor acidosis and a more invasive, migratory-active phenotype in vivo in different tumor entities. For example, Rolver et al. demonstrated that an acidic tumor microenvironment selects for a human pancreatic cancer stem cell subpopulation. Upon injection into mice, an aggressive growth and metastasis were observed compared to CTR, non-acidosis-treated cells [34]. Newly, we linked the senescence-like phenotype to that subpopulation. This is important when considering treatment options, as many therapeutics only aim to stop to tumor growth by targeting fast proliferating cells.

With Boyden chamber migration assays of whole cell populations, we could functionally affirm an increased migratory activity in LT NaHCO_3_ cells compared to CTR cells. However, subsequent live cell imaging analysis and tracking of single cells could not validate a general higher migratory phenotype under acidic conditions. Nonetheless, attachment and cell spreading were seriously impaired investigating the whole cell population, suggesting loss of cell-matrix contact and cell adhesion molecules as a general effect of acidosis treatment. This hypothesis was tested upon the generation of spheroids from acidosis-treated cells as well as control cells. We could show that more cells exited the spheroids generated from LT NaHCO_3_ than CTR cells, confirming a general effect of acidosis on cell-cell adhesion strength. Similar effects were also observed by Hofschrör et al. in melanoma, where extracellular protonation was shown to increase single-cell detachment from spheroids and to modulate tissue invasion [35]. We assume that only the subpopulation within the whole cell population, which we have characterized as a senescent phenotype, is accountable for migration and invasion, as tracking analysis of the C_12_FDG sorted subpopulations revealed a clear migratory phenotype for the LT NaHCO_3_ treated senescent C_12_FDG^high^ subpopulation. Here, we were able to prove that the acidosis-induced LT NaHCO_3_ C_12_FDG^high^ subpopulation of the tumor might be responsible for the formation of metastasis and thereby represents a promising target for new therapeutic strategies. In search for the underlying molecular mechanisms, we focused on enriched genes within the most prominent GSEA gene sets associated with cell migration. Subsequent qRT-PCR confirmed SEMA3C as one of the driver molecules promoting a migratory phenotype in the apparently senescent acidosis-treated LT NaHCO_3_ C_12_FDG^high^ subpopulation. Semaphorines include a group of about 20 membrane proteins which were originally discovered as guidance molecules for axons during nervous system wiring, but have nowadays been assigned a more versatile role, especially in cancer. While SEMA3B and SEMA3F have been identified as tumor suppressor molecules, SEMA3C, SEMA3E, and SEMA5C have been associated with a more migratory and invasive phenotype [36]. SEMA3C has also been discovered to drive EMT-related migration and invasion in prostate cancer [37]. Further, we revealed an increased mRNA expression of Caveolin-1 (CAV1) and Vascular endothelial growth factor receptor 1 (VEGFR-1/FLT1). CAV1 has also been assigned a contradictory role in cancer; however, the importance of the metastasis-promoting role in melanoma has been clearly defined [38]. CAV1 has been ascribed a role as a tumor suppressor, but on the other hand, high levels have been associated with enhanced metastasis in late-stage tumors. Lower levels have been found in melanocytes and primary tumor sides, while metastatic and invasive tumor cells correlate with high levels of CAV1 [39, 40]. Lobos-Gonzales et al. have described CAV1 as a marker of poor prognosis in melanomas, as preclinical models have shown the risk factor of CAV1 for post-surgery metastasis [40]. FLT1 (VEGFR-1) is a receptor tyrosine kinase and one of three members of the VEGF receptor family, the main receptors of vascular endothelial growth factors (VEGF), which promote angiogenesis to contribute to tumor growth and survival [41]. VEGFR-2 is best described out of the three receptors and has been assessed the main receptor for vasculogenesis and angiogenesis via MAPK pathway signaling. As VEGFR-3 has been discovered last, not much is known of its function, yet an involvement in cell migration has already been shown [41, 42]. VEGFR-1 or FLT1 is known as a specific receptor for VEGF-A, VEGF-B and Placental growth factor (PIGF). However, also a contrary effect on angiogenesis has been shown by Semaphorines and PlexinD1 (PLXND1)-mediated generation of soluble FLT1 (sFLT1), which acts as a decoy receptor and therefore can limit angiogenesis [41, 43–45]. Western blot analysis of FLT1 showed an alternative band pattern, indicating the expression of mature FLT1, being upregulated in the C_12_FDG^high^ subpopulation under acidic LT NaHCO_3_ conditions. Glycosylated, matured FLT1 has a 10-fold higher binding affinity for VEGF-A than VEGFR-2. Upon ligand binding, receptor homodimerization occurs as a substantial step for receptor activation [45]. Heuristic Online Phenotype Prediction (http://www.jurmo.ch/hopp/hopp_mpse.php), an online tool for predicting melanoma cell phenotypes according to specific gene expression, also revealed a strong correlation between FLT-1 expression and an invasive phenotype (retrieved on June 30^th^, 2024) [46].

FLT-1 and other VEGF receptors, as well as CAV1, are not only associated to cell migration but also with poor prognosis [22, 47–49]. Therefore, we analyzed melanoma prognostic marker S100B expression, which is significantly increased in the acidosis-treated strongly senescent subpopulation and therefore demonstrates the therapeutic relevance of the discovered isolated cell population in melanoma [25]. We hypothesize that the acidic tumor microenvironment drives plasticity and selects for a resistant phenotype, which can escape conventional therapy. This is supported by the finding that long-time acidosis-treated cells show a significantly lower number of apoptotic cells after treatment with chemotherapeutic topoisomerase II inhibitor etoposide than CTR cells treated at pH = 7.4 [7]. In the current study, we demonstrated that prolonged acidosis treatment leads to the formation of a small, senescence-like cell subpopulation within melanomas. This subpopulation exhibits migratory activity and high plasticity, capable of reinitiating proliferation in response to changes in the microenvironment. Consequently, this contributes to the progression of the disease. Current therapeutic approaches for melanoma are limited, as most of them struggle with the formation of resistances [28]. Developing new therapeutics is a tedious process, often lasting for decades, as research involves many setbacks. Normal in vitro cell culture experiments are performed at a pH of 7.4, which is not representing the acidic milieu of the tumor microenvironment. Hence, crucial information upon is missed. Therefore, we strongly suggest considering the tumor microenvironment when developing new treatment options.

## Methods

### Cell culture

Melanoma primary tumor cell line MEL-JUSO (CVCL_1403) and metastasis cell line SK-MEL-28 (CVCL_0526) were both cultivated in Dulbecco’s modified Eagle’s medium incl. 3.7 g/l NaHCO_3_ (DMEM, D6046, Sigma Aldrich, Steinheim, Germany) supplemented with 10% fetal bovine serum (FBS, PAN Biotech GmbH, Aidenbach, Germany) and 1% penicillin-streptomycin solution (P0781, Sigma Aldrich, Steinheim, Germany) at a pH of 7.4 in an incubator with humidified atmosphere at 8% CO_2_ and 37 °C. The cells were passaged every three to 4 days. For that, the cells were washed with Dulbecco’s phosphate-buffered saline (PBS, D8537, Sigma Aldrich, Steinheim, Germany) and detached using trypsin-EDTA solution (T4174, Sigma Aldrich, Steinheim, Germany). Trypsinization was stopped by adding culture media, and cells were pelleted by centrifugation (300 g, 4 min, RT). The supernatant was discarded, and the cells either passaged at a ratio of 1:5 (MEL-JUSO) or 1:3 (SK-MEL-28) or quantified using Neubauer counting chambers for further experimental use. Spheroids of the MEL-JUSO cell line were generated using the hanging drop method as described before [50]. Briefly, cells were counted and adjusted to 50,000/ml in their respective media. 20% of methocel (6 g methyl cellulose in 250 ml of the respective cell culture media) was added. For each spheroid, 25 µl of the cell suspension (500 cells/spheroids) was pipetted onto the cover of a petri dish, which was placed on top of the bottom half that was filled with 3 ml PBS to guarantee a humidified atmosphere. Incubation was carried out for 72 h before transfer of the formed spheroids into a polystyrene 12-well plate for further analysis. Mycoplasma contamination of the cell lines was excluded every 3 months using PhoenixDx Mycoplasma Mix (PCCSKU15209, Procomcure Biotech Germany GmbH, Hamburg, Germany).

### Acidosis treatment

The effects of an acidic tumor microenvironment were created in vitro by cultivating the respective cell lines at an extracellular pH (pH_e_) of 6.7. Therefore, DMEM powder medium (D5523, Sigma Aldrich, Steinheim, Germany) was dissolved according to the manufacturer’s instructions. The medium was sterilized by vacuum filtration (514-0328 P, VWR International, Radnor, USA). Subsequently, 10% FBS, 1% penicillin-streptomycin solution and 0.2 g/l sodium bicarbonate solution (S8761, Sigma Aldrich, Steinheim, Germany) as a CO2-dependent buffer system were added. The pH of 6.7 was adjusted in an incubator with a humidified atmosphere at 8% CO_2_ and 37 °C. Cells were cultivated by changing the media every 3–4 days or passaging them when 80–90% confluency was reached, according to the protocol described before, at a split ratio of 1:2. To capture the real tumor conditions as close as possible, long-time acidosis treatment was carried out at least 2 months prior to any experimental use. The pH stability of the media over a 72 h time frame was tested before the start of the long-time acidosis treatment (Supplementary Fig. 1).

### Analysis of mRNA expression

E.Z.N.A.® Total RNA Kit II (Omega Bio-Tek, Norcross, USA) was used to isolate total cellular RNA according to the manufacturer’s instructions. Complementary DNA (cDNA) was generated by reverse transcription reaction as previously described [51]. For real-time quantitative PCR (qRT-PCR) analysis, a LightCycler® 480 system (Roche, Basel, Switzerland) was used. Target gene expression was normalized to β-actin (ACTB) expression. Respective primer sequences are listed in Table 4.

**Table 4:** Oligonucleotides used for real-time PCR.

Gene	Forward primer	Reverse primer
ACTB	CTACGTCGCCCTGGACTTCGAGC	GATGGAGCCGCCGATCCACACGG
CAV1	GTCAACCGCGACCCTAAACA	GCCTTCCAAATGCCGTCAAA
CDKN1A	CGAGGCACCGAGGCACTCAGAGG	CCTGCCTCCTCCCAACTCATCCC
FLT1	AAATAAGCACACCACGCCCA	GCTTTGGTCAATTCGTCGCC
S100B	GGGAGGGAGACAAGCACAAG	TCGCCGTCTCCATCATTGTC
SEMA3C	AACAGATGAAGACGGCCCAG	GTTCCAGGGCGAGGATATGG

### Western blot analysis

Total protein isolates (RIPA lysates) for western blot analysis were generated as described before [52]. RIPA lysates (20–30 µg per lane) were loaded onto a 15% sodium dodecyl sulfate polyacrylamide gel and separated via electrophoresis (SDS-PAGE). The separated proteins were then transferred onto a polyvinylidene difluoride membrane (Bio-Rad Laboratories, Inc., Hercules, USA) by a semi-dry blotting apparatus (Biometra GmbH, Göttingen, Germany). The membranes were blocked in 5% non-fat dried milk (70166, Sigma Aldrich, Steinheim, Germany) in TBS-T for 1 h at room temperature (RT). Primary antibodies against p21 (1:1000, ab109199, Abcam, Berlin, Germany), VEGFR1/FLT1 (1:1000, NBP3-11864; Novus Biologicals, Toronto, Canada), S100b (1:1000, Z0311, Agilent Dako, Santa Clara, USA) and β-actin (1:5000, A5441, Sigma Aldrich, Steinheim, Germany) were incubated overnight at 4 °C. Secondary antibodies anti-rabbit IgG, HRP-linked (1:2000, #7074, Cell Signaling Technology, Danvers, USA) and anti-mouse IgG, HRP-linked (1:2000, #7076, Cell Signaling Technology, Danvers, USA) were incubated for 1 h at RT. Proteins were detected by Clarity Western ECL Substrate (#1705061, Bio-Rad Laboratories, Inc., Hercules, USA) and visualized by a ChemoStar Chemiluminescence Imager (Intas, Goettingen, Germany). For densiometric quantification, LabImage software (Version 4.2.3, Kapelan Bio-Imaging GmbH, Leipzig, Germany) was used.

### Senescence-associated β-galactosidase staining

Detection of senescence-associated β-galactosidase activity (SA β-Gal) was carried out using the Senescence β-Galactosidase Staining Kit (#9860, Cell Signaling Technology, Danvers, USA) according to the manufacturer’s instructions. As a positive control, the cells were treated with 100 µM etoposide (E1383, Sigma Aldrich, Steinheim, Germany) for 48 h as described before [11]. Reintroduction of physiological pH (RE) of LT-acidosis-treated melanoma cells was done by cultivating cells in the respective control media at pH 7.4 for 96 h before staining was carried out. Images of the stained cells were obtained using an inverted microscope system (IX83 Inverted Microscope, Olympus Life Sciences, Tokyo, Japan). The percentage of senescent β-galactosidase-positive (blue) cells was determined manually with the help of ImageJ software (Version 2.14.0/1.54 f) by counting the stained cells in comparison to the total number of cells in each picture.

### Luciferase reporter gene assay

Transfection of plasmid DNA into cells was completed using Lipofectamine^TM^ LTX & PLUS^TM^ Reagent (A12621, Invitrogen by Thermo Fisher Scientific, Waltham, USA) according to the manufacturer’s instructions. We co-transfected the cells with pGreenFire p53 Lenti-Reporter (kindly provided by Prof. Dr. Svenja Meierjohann, University of Wuerzburg) with wild-type Renilla reporter pRL-TK. pGL3basic was used as a control. A Dual-Luciferase® Reporter (DLR^TM^) Assay System (E1960, Promega, Madison, USA) was used for analysis as described before [53]. For Firefly and Renilla signal measurement, a LUC Centro XS^3^ Microplate reader (Berthold Technologies, Bad Wildbad, Germany) was used.

### Clonogenic assay

Analysis of clonogenicity of melanoma cells was performed by plating 100 cells into a well of a polystyrene 6-well plate (CLS3506, Sigma Aldrich, Steinheim, Germany) and incubating them for 14 d in a humidified atmosphere with 8% CO_2_ at 37 °C. Control cells (CTR) and cells reintroduced (RE) to physiological pH 7.4 were incubated in media as described in *Cell Culture*. LT-acidosis (LT NaHCO_3_) treated cells at pH 6.7 were incubated in media as described in *Acidosis Treatment*. Cells were fixed and stained in a mixture of 6% glutaraldehyde solution (G6257, Sigma Aldrich, Steinheim, Germany) and 0.36% crystal violet solution (V5265, Sigma Aldrich, Steinheim, Germany) for 30 min at RT. Afterwards, the cells were washed three times with water. Images of the stained cells were obtained using an inverted microscope system (IX83 Inverted Microscope, Olympus Life Sciences, Tokyo, Japan). The number of colonies and their sizes were quantified manually with the help of the Count and Measure Tool of CellSens Dimension Software (Version 2.3, Olympus Life Sciences, Tokyo, Japan).

### Boyden chambers

To analyze the directed migration of melanoma cells, Boyden Chamber assays were performed as described before in [54].

### Flow cytometry of fluorescent β-galactosidase substrate C_12_FDG

The β-galactosidase substrate, 5-dodecanoylaminofluorescein di-β-D-galactopyranoside (C_12_FDG, D2893, Thermo Fisher Scientific, Waltham, USA) was used for the detection of SA-β-Gal activity in melanoma by flow cytometry as described before [15]. Briefly, the cells (3.5 × 105 per well) were incubated with 100 nM bafilomycin A1 (19-148, Sigma Aldrich, Steinheim, Germany) for 1 h to adjust lysosomal pH to 6, before 33 µM C_12_FDG was added for further incubation of 1 h. After that, the cells were detached according to the protocol described in paragraph *Cell Culture* and the pellet resuspended in a 2% bovine serum albumin (BSA, A7906, Sigma Aldrich, Steinheim, Germany) in PBS solution for measurement on a FACS-LSRFortessa^TM^ X-20 (BD Biosciences, Franklin Lakes, USA). Analyses were performed using BD FACSDiva^TM^ Software (Version 8.0, BD Biosciences, Franklin Lakes, USA) and FlowJo^TM^ Software (Version 10, FlowJo LLC, Ashland, USA). Fluorescent activated cell sorting of primary tumor cell line MEL-JUSO was carried on a MoFlo^TM^ XDP Cell Sorter (Beckman Coulter, Brea, USA). For that, the lowest and highest two percent of C_12_FDG stained CTR (pH 7.4) and LT NaHCO_3_ (pH 6.7) cells were isolated into two fractions (negative/high) for further use.

### Time-lapse imaging and cell tracking

Time-lapse imaging of MEL-JUSO and SK-MEL-28 cell lines, as well as MEL-JUSO sorted C_12_FDG subpopulations, was performed on an IX83 Inverted Microscope (Olympus Life Sciences, Tokyo, Japan) with an Incubator OL IX73/IX83 cell vivo-3 Variant 2, Heating Unit 2000, TempController 2000-2 cell vivo design and CO_2_-Controller 200 cell vivo design (all from PeCon GmbH, Erbach, Germany) to imitate cell culture conditions described in the paragraphs *Cell Culture* and *Acidosis Treatment* for a 96 h analyzation period. Each time-lapse series was processed using differential contrast enhancement and a rank smoothing filter before using the CellSens software’s Count and Measure and Object Tracking Tool (Olympus Life Sciences, Tokyo, Japan) solutions to track cell migration, count cells and determine their attachment time.

### RNA-sequencing library preparation, data preprocessing and analysis

Isolation of Total RNA samples was done as described. All RNA samples were examined for integrity and purity by TapeStation 4200 (Agilent, Santa Clara, USA). Library preparation was performed with three biological replicates using the TruSeq® Stranded Total RNA Library Prep Human/Mouse/Rat Kit according to the manufacturer’s instructions (20020596, Illumina Inc., San Diego, USA). The resulting libraries were checked for size (200–500 bp) by TapeStation 4200 (Agilent, Santa Clara, USA) using High Sensitivity DNA Kit (5067-4626, Agilent, Santa Clara, USA) and concentration by a Qubit 4 Fluorometer (Thermo Fisher Scientific, Waltham, USA). Sequencing was performed on a NovaSeq 6000 SP Sequencing System with a paired-end module (Illumina, San Diego, USA) according to the manufacturer’s instructions. The samples were sequenced from each side of a fragment approximately 100 bp long, with an average number of 40 million reads per sample with regard to the sequencing of the selected subpopulations and 20 million reads with regard to the total population. After quality check using FastQC (Version 0.11.9, Babraham Bioinformatics—FastQC A Quality Control Tool for High Throughput Sequence Data, accessed on May 17th, 2022), paired-end reads were aligned to the human genome (GRCh38.p5, release 24) using the STAR alignment software (Version 2.7.9a) [55]. After mapping, only reads that mapped to a single unique location were considered for further analysis. The mapped reads were then used to generate a count table using the featureCounts software (Version 2.0.1) [56]. The raw reads were filtered, normalized and visualized using R (Version 4.3.2, The R Foundation for Statistical Computing, Vienna, Austria) [57]. The DESeq2 package (Version 1.42.1) was used for logarithmic transformation of the data and for data exploration [58]. Differential expression analysis was performed using the DESeq2 standard approach. Adjusted *p*-values were calculated using the Benjamini-Hochberg method within DESeq2. Gene annotations were added to the results files using Ensemble data. Differentially expressed genes with an adjusted *p*-value < 0.1 were regarded as statistically significant. Functional data analysis was performed using the Gene Set Enrichment Analysis Tool (GSEA, Version 4.2.3) [59, 60] and the multi-dimensional enrichment tool mitch [17]. Enrichment analysis using C2 Canonical pathways, C5 GO Biological Processes, Hallmark gene sets, and senescent gene sets from MSigDB (Version 2023) [60, 61] was performed with classical weighting, the Signal2Noise metric and 1000 permutations of gene sets. Mitch analysis was conducted along the effect size of gene sets. Subsequent networks of the differentially expressed genes were produced by Cytoscape (Version 3.9.1) [62]. Enrichment results were considered significant with FDR < 0.25.

### Statistical analysis

Statistical analysis of experimental results was executed using GraphPad Prism 10 software (Version 10.1.1; GraphPad Software Inc., San Diego, CA, USA). If not stated otherwise, all results are normalized to the respective control and shown as mean ± SEM (range). Comparison between the two groups was made by using Student’s unpaired *t*-test. Comparison between three groups was made by using one-way ANOVA followed by Tukey’s HSD post hoc test, respectively. Comparison between groups with more variables was made by using or two-way ANOVA. All experiments were repeated at least three times in an independent manner. A critical *p*-value of *p* < 0.05 was considered statistically significant if not stated otherwise. No indication between groups implies no statistical significance.

## Supplementary information

Suppl. Tables

Suppl. Figures

## Data Availability

The RNA-sequencing data used in this study have been deposited in the NCBI BioProject database (https://www.ncbi.nlm.nih.gov/bioproject/) and can be accessed with the BioProject accession number PRJNA1200919. Source data for the figures are provided with the paper.
