# Supplementary material for: Acidic melanoma microenvironment selects for a senescence-like but also migratory-active subpopulation driving metastatic disease
Source: Cell Death Discov. 2025 Oct 20;11:469. doi: 10.1038/s41420-025-02806-0 (PMC12537852; doi:10.1038/s41420-025-02806-0)
Supplement: Supplementary file 1 — Suppl. Tables [file 41420_2025_2806_MOESM1_ESM.docx]

# SUPPLEMENT TABLES

**Supplement Table 1: Top20 of significant enriched gene sets in LT NaHCO3 C12FDG^high^ compared to CTR NaHCO3 C12FDG^high^**

| **Gene set** | **Size** | **NES** | **FDR** |
| --- | --- | --- | --- |
| [GOBP_ANTIGEN_PROCESSING_AND_PRESENTATION_OF_EXOGENOUS_ANTIGEN](http://www.gsea-msigdb.org/gsea/msigdb/human/geneset/GOBP_ANTIGEN_PROCESSING_AND_PRESENTATION_OF_EXOGENOUS_ANTIGEN) | 47 | 2.85 | 0 |
| [GOBP_NEGATIVE_REGULATION_OF_CHEMOTAXIS](http://www.gsea-msigdb.org/gsea/msigdb/human/geneset/GOBP_NEGATIVE_REGULATION_OF_CHEMOTAXIS) | 62 | 2.61 | 0 |
| [GOBP_ANTIGEN_PROCESSING_AND_PRESENTATION_OF_EXOGENOUS_PEPTIDE_ANTIGEN](http://www.gsea-msigdb.org/gsea/msigdb/human/geneset/GOBP_ANTIGEN_PROCESSING_AND_PRESENTATION_OF_EXOGENOUS_PEPTIDE_ANTIGEN) | 38 | 2.58 | 0 |
| [GOBP_PEPTIDE_ANTIGEN_ASSEMBLY_WITH_MHC_PROTEIN_COMPLEX](http://www.gsea-msigdb.org/gsea/msigdb/human/geneset/GOBP_PEPTIDE_ANTIGEN_ASSEMBLY_WITH_MHC_PROTEIN_COMPLEX) | 18 | 2.54 | 0 |
| [GOBP_ANTIGEN_PROCESSING_AND_PRESENTATION_OF_EXOGENOUS_PEPTIDE_ANTIGEN_VIA_MHC_CLASS_II](http://www.gsea-msigdb.org/gsea/msigdb/human/geneset/GOBP_ANTIGEN_PROCESSING_AND_PRESENTATION_OF_EXOGENOUS_PEPTIDE_ANTIGEN_VIA_MHC_CLASS_II) | 29 | 2.52 | 0 |
| [GOBP_EXTERNAL_ENCAPSULATING_STRUCTURE_ORGANIZATION](http://www.gsea-msigdb.org/gsea/msigdb/human/geneset/GOBP_EXTERNAL_ENCAPSULATING_STRUCTURE_ORGANIZATION) | 308 | 2.46 | 0 |
| [GOBP_NEGATIVE_REGULATION_OF_AXONOGENESIS](http://www.gsea-msigdb.org/gsea/msigdb/human/geneset/GOBP_NEGATIVE_REGULATION_OF_AXONOGENESIS) | 64 | 2.43 | 0 |
| [GOBP_ANTIGEN_PROCESSING_AND_PRESENTATION_OF_PEPTIDE_OR_POLYSACCHARIDE_ANTIGEN_VIA_MHC_CLASS_II](http://www.gsea-msigdb.org/gsea/msigdb/human/geneset/GOBP_ANTIGEN_PROCESSING_AND_PRESENTATION_OF_PEPTIDE_OR_POLYSACCHARIDE_ANTIGEN_VIA_MHC_CLASS_II) | 33 | 2.38 | 0 |
| [GOBP_SEMAPHORIN_PLEXIN_SIGNALING_PATHWAY](http://www.gsea-msigdb.org/gsea/msigdb/human/geneset/GOBP_SEMAPHORIN_PLEXIN_SIGNALING_PATHWAY) | 44 | 2.38 | 0 |
| [GOBP_POSITIVE_REGULATION_OF_PEPTIDYL_TYROSINE_PHOSPHORYLATION](http://www.gsea-msigdb.org/gsea/msigdb/human/geneset/GOBP_POSITIVE_REGULATION_OF_PEPTIDYL_TYROSINE_PHOSPHORYLATION) | 172 | 2.36 | 0 |
| [GOBP_NEURON_PROJECTION_EXTENSION_INVOLVED_IN_NEURON_PROJECTION_GUIDANCE](http://www.gsea-msigdb.org/gsea/msigdb/human/geneset/GOBP_NEURON_PROJECTION_EXTENSION_INVOLVED_IN_NEURON_PROJECTION_GUIDANCE) | 38 | 2.35 | 0 |
| [GOBP_NEGATIVE_CHEMOTAXIS](http://www.gsea-msigdb.org/gsea/msigdb/human/geneset/GOBP_NEGATIVE_CHEMOTAXIS) | 47 | 2.34 | 0 |
| [GOBP_RENAL_SYSTEM_PROCESS](http://www.gsea-msigdb.org/gsea/msigdb/human/geneset/GOBP_RENAL_SYSTEM_PROCESS) | 123 | 2.30 | 0 |
| [GOBP_MESENCHYMAL_CELL_MIGRATION](http://www.gsea-msigdb.org/gsea/msigdb/human/geneset/GOBP_MESENCHYMAL_CELL_MIGRATION) | 60 | 2.30 | 0 |
| [GOBP_INTEGRIN_MEDIATED_SIGNALING_PATHWAY](http://www.gsea-msigdb.org/gsea/msigdb/human/geneset/GOBP_INTEGRIN_MEDIATED_SIGNALING_PATHWAY) | 108 | 2.29 | 0 |
| [GOBP_REGULATION_OF_NEUROTRANSMITTER_RECEPTOR_ACTIVITY](http://www.gsea-msigdb.org/gsea/msigdb/human/geneset/GOBP_REGULATION_OF_NEUROTRANSMITTER_RECEPTOR_ACTIVITY) | 60 | 2.29 | 0 |
| [GOBP_NEGATIVE_REGULATION_OF_AXON_EXTENSION](http://www.gsea-msigdb.org/gsea/msigdb/human/geneset/GOBP_NEGATIVE_REGULATION_OF_AXON_EXTENSION) | 45 | 2.28 | 0 |
| [GOBP_REGULATION_OF_ACUTE_INFLAMMATORY_RESPONSE](http://www.gsea-msigdb.org/gsea/msigdb/human/geneset/GOBP_REGULATION_OF_ACUTE_INFLAMMATORY_RESPONSE) | 42 | 2.28 | 0 |
| [GOBP_HOMOPHILIC_CELL_ADHESION_VIA_PLASMA_MEMBRANE_ADHESION_MOLECULES](http://www.gsea-msigdb.org/gsea/msigdb/human/geneset/GOBP_HOMOPHILIC_CELL_ADHESION_VIA_PLASMA_MEMBRANE_ADHESION_MOLECULES) | 164 | 2.28 | 0 |
| [GOBP_NEGATIVE_REGULATION_OF_SMOOTH_MUSCLE_CELL_MIGRATION](http://www.gsea-msigdb.org/gsea/msigdb/human/geneset/GOBP_NEGATIVE_REGULATION_OF_SMOOTH_MUSCLE_CELL_MIGRATION) | 28 | 2.28 | 0 |

**Supplement Table 2: Gene sets used for investigation of migration phenotype selected from all significant enriched gene sets in LT NaHCO_3_ C_12_FDG^high^**

| **Gene sets** |
| --- |
| HALLMARK_EPITHELIAL_MESENCHYMAL_TRANSITION |
| REACTOME_DEGRADATION_OF_EXTRACELLULAR_MATRIX |
| KEGG_ECM_RECEPTOR_INTERACTION |
| REACTOME_EXTRACELLULAR_MATRIX_ORGANISATION |
| KEGG_FOCAL_ADHESION |
| REACTOME_INTEGRIN_CELL_SURFACE_INTERACTIONS |
| REACTOME_NON_INTEGRIN_MEMBRANE_ECM_INTERACTIONS |
| REACTOME_COLLAGEN_DEGRADATION |
| KEGG_CELL_ADHESION_MOLECULES_CAMS |
| HALLMARK_APICAL_JUNCTIONS |
| REACTOME_ECM_PROTEOGLYCANS |
| NABA_ECM_GLYCOPROTEINS |
| REACTOME_MET_ACTIVATES_PTK2_SIGNALING |
| WP_FOCAL_ADHESION_PI3AKTMTORSIGNALING_PATHWAY |
| WP_STRIATED_MUSCLE_CONTRACTION_PATHWAY |
| GOBP_NEURON_PROJECTION_EXTENSION_INVOLVED_IN_NEURON_PROJECTION_GUIDANCE |
