# Supplementary material for: Acidic melanoma microenvironment selects for a senescence-like but also migratory-active subpopulation driving metastatic disease
Source: Cell Death Discov. 2025 Oct 20;11:469. doi: 10.1038/s41420-025-02806-0 (PMC12537852; doi:10.1038/s41420-025-02806-0)
Supplement: Supplementary file 2 — Suppl. Figures [file 41420_2025_2806_MOESM2_ESM.pdf]

Supplement Fig. 1

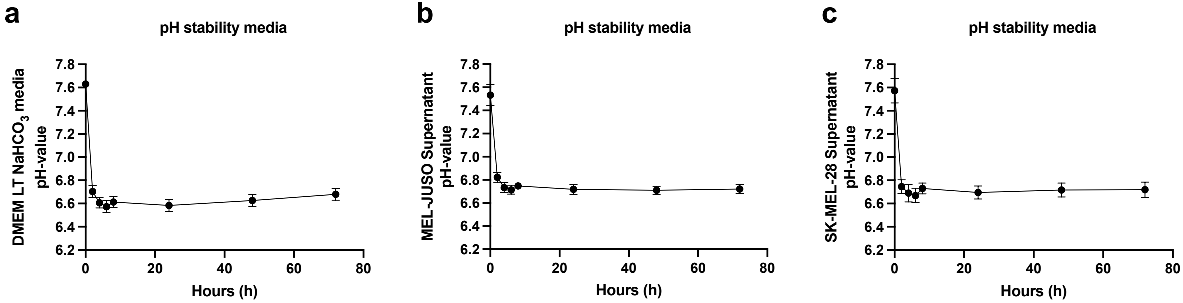

Supplement Fig. 1: pH stability test. a The pH value of the DMEM LT $NaHCO_3$ media was measured at time points 2 h, 4 h, 6 h, 8 h, 24 h, 48 h and 72 h post incubation in an incubator at 8 % CO_2 and 37 °C (n=3). **b** The pH value of the supernatant of MEL-JUSO cells was measured at time points 2 h, 4 h, 6 h, 8 h, 24 h, 48 h and 72 h (n=5). **c** The pH value of the supernatant of SK-MEL-28 cells was measured at time points 2 h, 4 h, 6 h, 8 h, 24 h, 48 h and 72 h (n=5). Results are shown as mean ± SEM (range).

Supplement Fig. 2

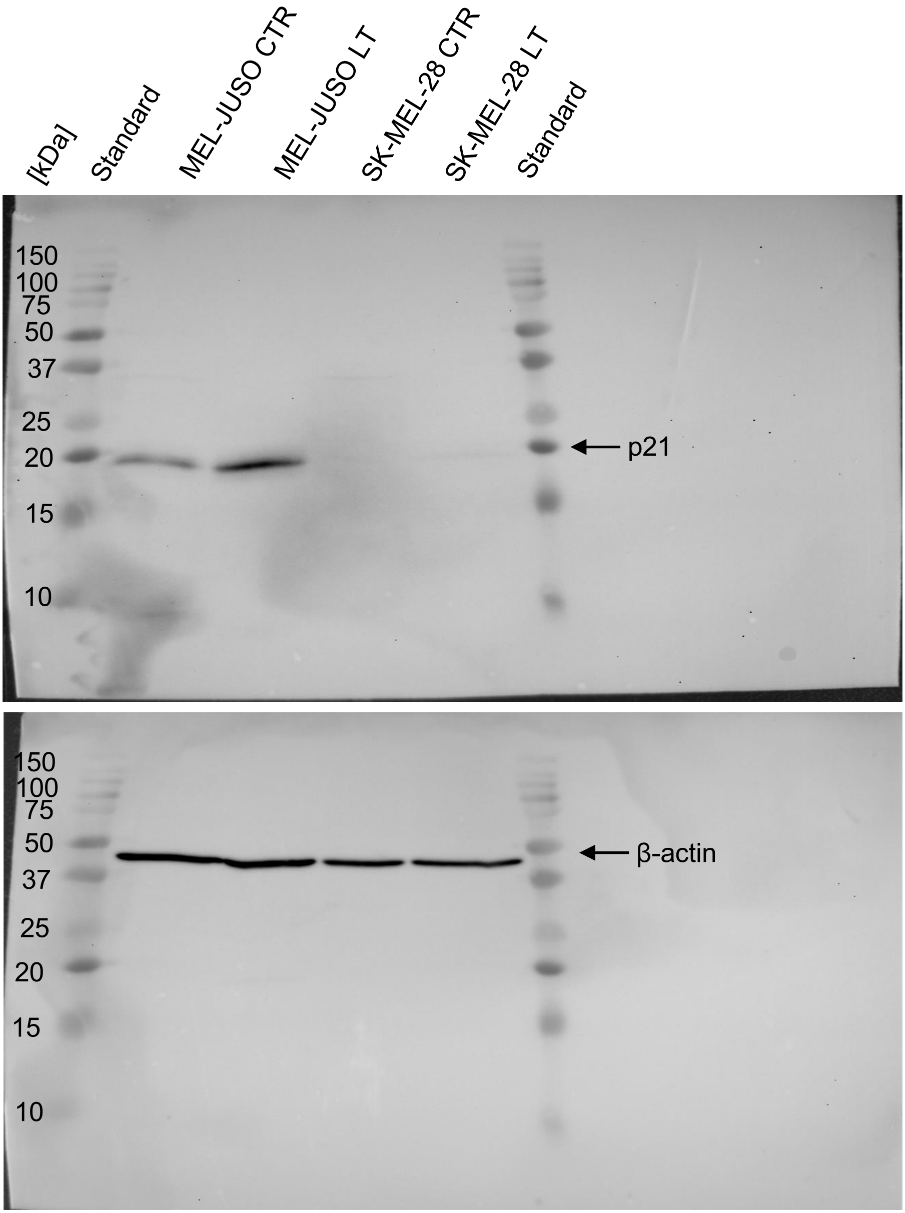

Supplement Fig. 2: Uncropped western blot for p21. See cropped blot Fig. 1d.

Supplement Fig. 3

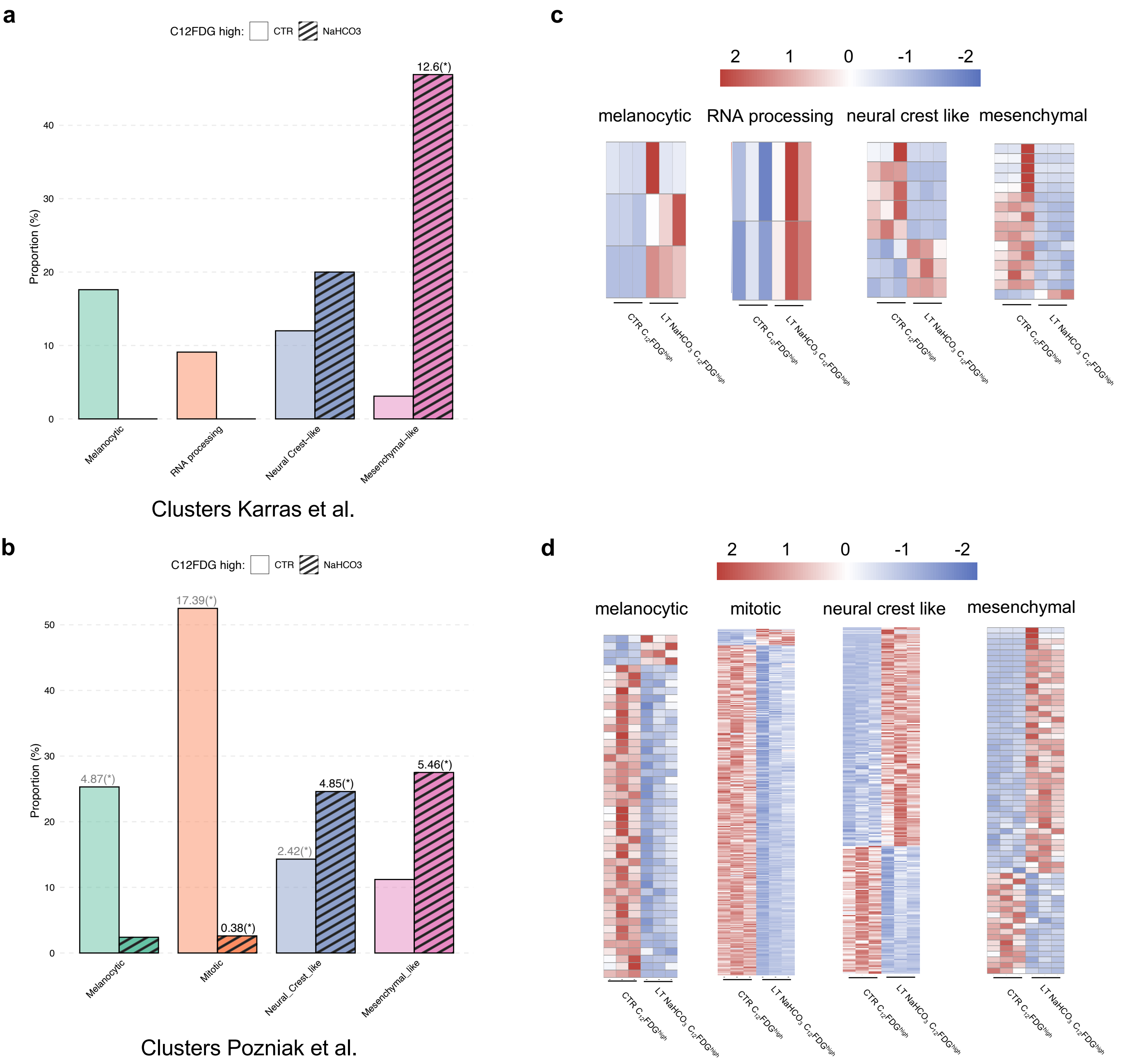

Supplement Fig. 3: Overrepresentation analyses of upregulated genes within melanoma signature genes. Barplots depicting the proportion of overlaps between upregulated genes in either CTR $C_{12}FDG^{high}$ (transparent/light) or LT $NaHCO_3$ $C_{12}FDG^{high}$ (opaque/dark) with genes assigned to the different melanoma signature clusters by **(a)** Karras et al. [18] or **(b)** Pozniak et al. [19]. For statistically significant results (*p-value < 0.01: Fisher's exact test), the odds ratio is given above individual bars, representing the strengh of the association between gene upregulation and membership in the gene signature. **c** Expression status of the respective marker genes of the melanocytic, RNA processing, neural crest like and mesenchymal cell type clusters from Karras et al. in CTR $C_{12}FDG^{high}$ and LT $NaHCO_3$ $C_{12}FDG^{high}$ subpopulations based on the scaled normalized RNA-seq count data. **d** Expression status of the respective marker genes of the melanocytic, mitotic, neural crest like and mesenchymal cell type clusters from Pozniak et al. in CTR $C_{12}FDG^{high}$ and LT $NaHCO_3$ $C_{12}FDG^{high}$ subpopulations based on the scaled normalized RNA-seq count data.

Supplement Fig. 4

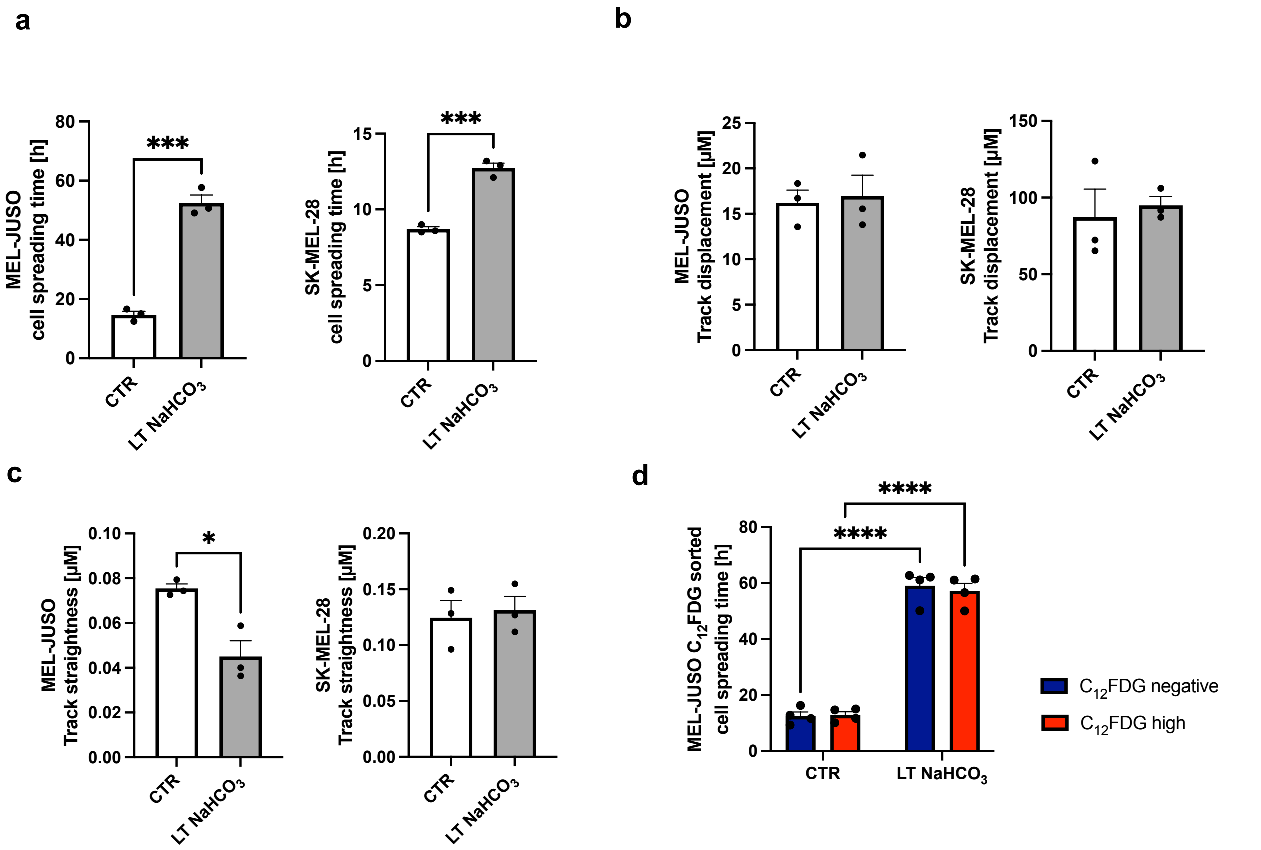

Supplement Fig. 4: Analysis of migratory phenotype after LT acidosis treatment of melanoma cells. a Time measurement (h) until cells were attached and spreaded of MEL-JUSO and SK-MEL-28 cell lines after LT acidosis treatment at pH 6.7 (LT $NaHCO_3$) compared to cells cultivated at pH 7.4 (CTR). Results are shown as mean ± SEM (range). Statistical analysis was made using Student's unpaired t-test. **b** Live imaging analysis. Track displacement of MEL-JUSO and SK-MEL-28 cell lines after LT acidosis treatment at pH 6.7 (LT $NaHCO_3$) compared to cells cultivated at pH 7.4 (CTR) after 96h. Results are shown as mean ± SEM (range). Statistical analysis was made using Student's unpaired t-test. **c** Live imaging analysis. Track straightness of MEL-JUSO and SK-MEL-28 cell lines after LT acidosis treatment at pH 6.7 (LT $NaHCO_3$) compared to cells cultivated at pH 7.4 (CTR) after 96h. Results are shown as mean ± SEM (range). Statistical analysis was made using Student's unpaired t-test. **d** Time measurement (h) until MEL-JUSO subpopulations after C_{12}FDG FACS were attached and spreaded. Results are shown as mean ± SEM (range). Statistical analysis was made using two-way ANOVA.

Supplement Fig. 5

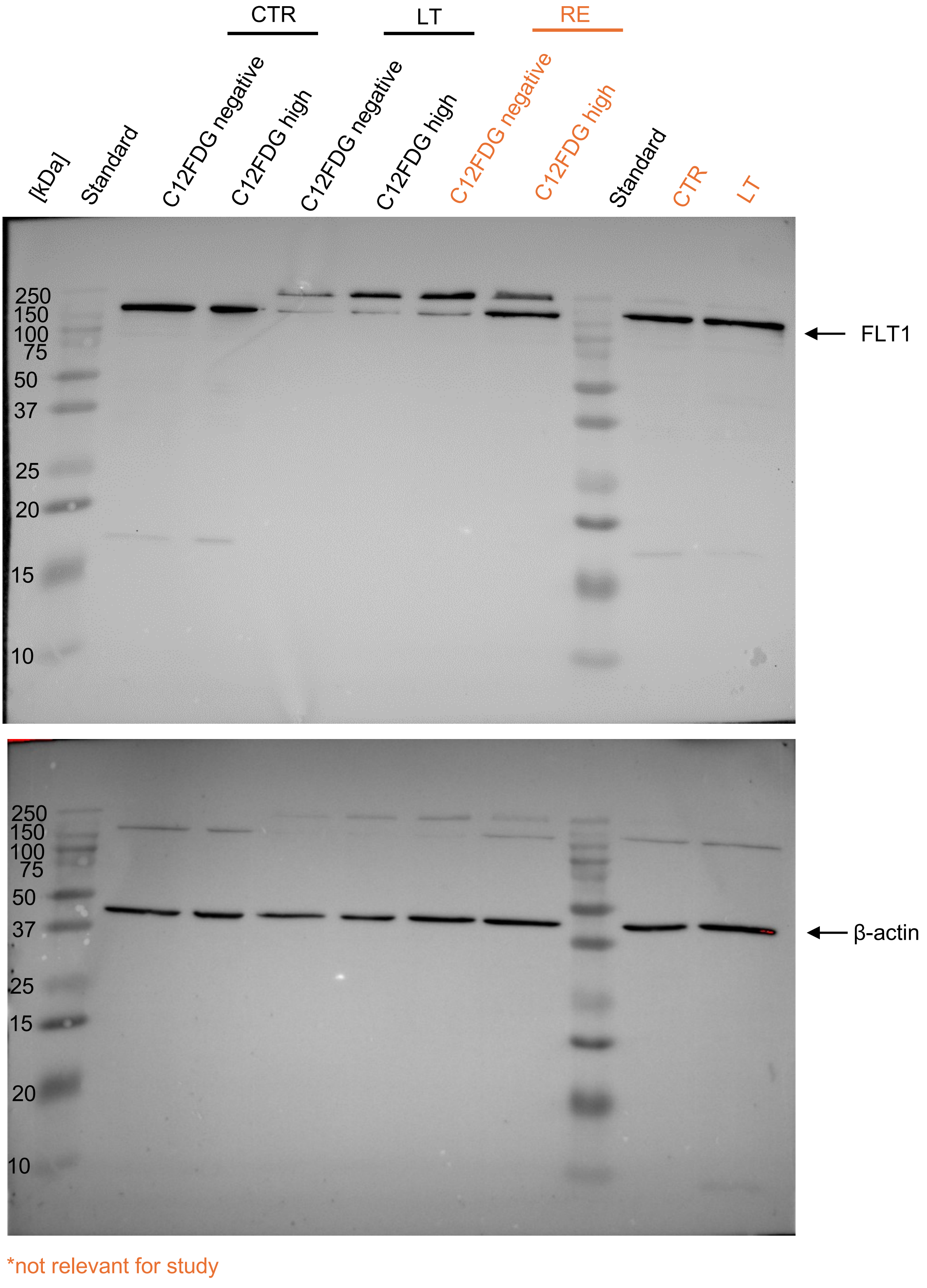

Supplement Fig. 5: Uncropped western blot for FLT1. See cropped blot Fig. 6e.

Supplement Fig. 6

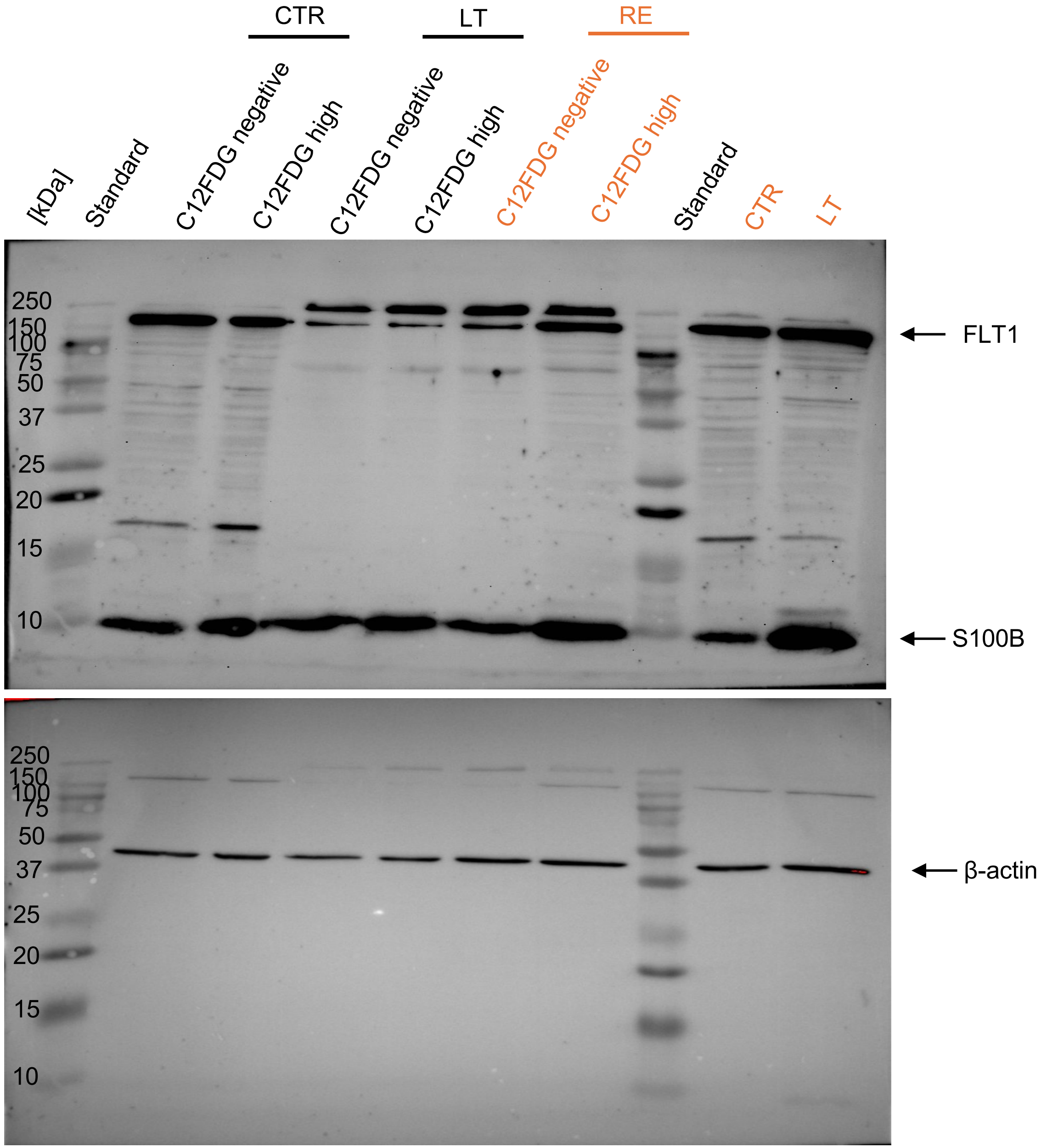

*not relevant for study

Supplement Fig. 6: Uncropped western blot for S100B. See cropped blot Fig. 6e.
